# FOXF1 transcription factor promotes lung regeneration after partial pneumonectomy

**DOI:** 10.1038/s41598-017-11175-3

**Published:** 2017-09-06

**Authors:** Craig Bolte, Hannah M. Flood, Xiaomeng Ren, Sajjeev Jagannathan, Artem Barski, Tanya V. Kalin, Vladimir V. Kalinichenko

**Affiliations:** 1 Center for Lung Regenerative Medicine, Cincinnati Children’s Research Foundation, Cincinnati, Ohio, USA; 2 Division of Pulmonary Biology, Cincinnati Children’s Research Foundation, Cincinnati, Ohio, USA; 3 Division of Allergy and Immunology, Director of Epigenomics Data Analysis Core, Cincinnati Children’s Research Foundation, Cincinnati, Ohio, USA

## Abstract

FOXF1, a member of the forkhead box family of transcription factors, has been previously shown to be critical for lung development, homeostasis, and injury responses. However, the role of FOXF1 in lung regeneration is unknown. Herein, we performed partial pneumonectomy, a model of lung regeneration, in mice lacking one *Foxf1* allele in endothelial cells (*PDGFb-iCre/Foxf1* ^*fl*/+^ mice). Endothelial cell proliferation was significantly reduced in regenerating lungs from mice deficient for endothelial *Foxf1*. Decreased endothelial proliferation was associated with delayed lung regeneration as shown by reduced respiratory volume in *Foxf1*-deficient lungs. FACS-sorted endothelial cells isolated from regenerating *PDGFb-iCre/Foxf1* ^*fl*/+^ and control lungs were used for RNAseq analysis to identify FOXF1 target genes. *Foxf1* deficiency altered expression of numerous genes including those regulating extracellular matrix remodeling (*Timp3*, *Adamts9*) and cell cycle progression (*Cdkn1a*, *Cdkn2b, Cenpj*, *Tubb4a*), which are critical for lung regeneration. Deletion of *Foxf1* increased *Timp3* mRNA and protein, decreasing MMP14 activity in regenerating lungs. ChIPseq analysis for FOXF1 and histone methylation marks identified DNA regulatory regions within the *Cd44*, *Cdkn1a*, and *Cdkn2b* genes, indicating they are direct FOXF1 targets. Thus FOXF1 stimulates lung regeneration following partial pneumonectomy via direct transcriptional regulation of genes critical for extracellular matrix remodeling and cell cycle progression.

## Introduction

Lung regeneration plays an important role in lung repair after injury. It is reliant upon proliferation of multiple cell types in the lung, including endothelium, epithelium, and fibroblasts, as well as remodeling of the extracellular matrix. Lung regeneration following injury progresses via an initial inflammatory response during which macrophages clear the tissue of cellular debris. This process continues through cellular proliferation when existing cells and progenitors act to repopulate cells lost during injury, followed by tissue maturation in which newly formed cells achieve a differentiated phenotype^1^. Signaling pathways critical for lung regeneration include FGF, EGF, WNT, and NOTCH. In addition, HDACs, miRNAs, ELASTIN, and MMP14 have been shown to regulate lung regeneration^2, 3^. Partial pneumonectomy (PNX) has been used as a therapeutic and investigational tool for several decades. Following PNX the remaining lung increases in size to compensate for loss of volume and respiratory capacity. Much has been learned about the triggers and mechanisms regulating pulmonary regeneration. However, the role of endothelial cells in post-PNX lung growth remains incompletely characterized. Endothelial cells are essential for alveolar structure as well as to mediate gas exchange between the alveoli and circulatory system. Two divergent processes have been proposed to stimulate post-PNX lung growth. Lung volume may be recuperated by increasing the size of individual alveoli, leading to a simpler lung structure while increasing gas exchange surface area^4^. The alternative process is to increase the number of alveoli, which maintains a consistent pulmonary morphology while expanding lung volume and surface area for gas exchange^4^. Both processes of post-PNX lung growth involve a period of rapid cell proliferation including endothelial and epithelial cells as both are essential for formation of alveoli to restore respiratory capacity after resection. While endothelial cells stimulate epithelial proliferation and induce lung repair and regeneration via MMP14-mediated release of EGF ligands^3^, the transcriptional mechanisms regulating endothelial cells during lung regeneration remain largely unknown.

Foxf1 transcription factor (previously known as HFH-8 or Freac1) is known to be an essential mediator of lung development and embryonic angiogenesis*. Foxf1* ^*−/−*^ mice are embryonic lethal due to abnormal development of yolk sac, vasculature, and allantois^5–8^. Haploinsufficiency for *Foxf1* causes severe lung malformations and inhibits development of pulmonary capillaries during embryonic and early postnatal periods^7^. Heterozygous deletions and point mutations in the *FOXF1* gene locus were found in more than 40% of patients with alveolar-capillary dysplasia with misalignment of pulmonary veins (ACD/MPV)^9^. Conditional deletion of *Foxf1* from endothelial cells is sufficient to cause embryonic lethality due to severely underdeveloped vasculature in the yolk sac and placenta as well as the loss of VEGF receptors, FLK1 and FLT1 from the surface of endothelial cells^10^. In adult mice, endothelial-specific deletion of both *Foxf1* alleles results in uniform lethality due to pulmonary hemorrhage, massive inflammatory cell infiltration, and pulmonary edema, resulting from disruption of adherens junctions and loss of endothelial barrier function^11^. Deletion of only one *Foxf1* allele from endothelial cells conferred greater susceptibility to butylated hydroxytoluene- (BHT) or lipopolysaccharide- (LPS) induced lung injury^11^. FOXF1 regulates expression of genes critical for maintenance of the endothelial barrier, such as VE-cadherin (*Cdh5*) and sphingosine 1-phosphate receptor 1 (*S1pr1*)^11^. *Foxf1* ^+/−^ mice have also been shown to be more susceptible to chemically induced lung^12, 13^ and liver injury^14^. While these studies demonstrate a critical role for FOXF1 in lung injury and inflammation, molecular mechanisms regulated by FOXF1 during proliferative responses induced by PNX remain uncharacterized.

In the present study, a PNX model was used to investigate the role of FOXF1 in lung regeneration. Since deletion of both *Foxf1* alleles in endothelial cells is lethal^11^, a tamoxifen-inducible *Cre* (*PDGFb-iCre*) was used to cause *Foxf1* haploinsufficiency in endothelial cells prior to PNX surgery. *PDGFb-iCre/Foxf1* ^*fl*/+^ mice exhibited delayed lung regeneration associated with diminished proliferative capacity of endothelial cells. Our study shows that FOXF1 transcriptionally regulates *Timp3*, *Cdkn1a*, *Cdkn2b*, and *Cd44*, all of which are critical for extracellular matrix remodeling and endothelial proliferation. This study demonstrates that FOXF1 induces lung regeneration and reestablishment of normal alveolar structure after PNX.

## Methods

### Ethics statement

All animal experiments and protocols used in the study were approved by and carried out in accordance with the guidelines of the Cincinnati Children’s Research Foundation Institutional Animal Care and Use Committee (IACUC) and the NIH IACUC Guidebook. All experiments were covered under our animal protocol (IACUC2016-0038). The Cincinnati Children’s Research Foundation Institutional Animal Care and Use Committee is an AAALAC and NIH accredited institution (NIH Insurance #8310801).

### Generation of mice with conditional deletion of ***Foxf1*** from endothelial cells

**Figure 1:**
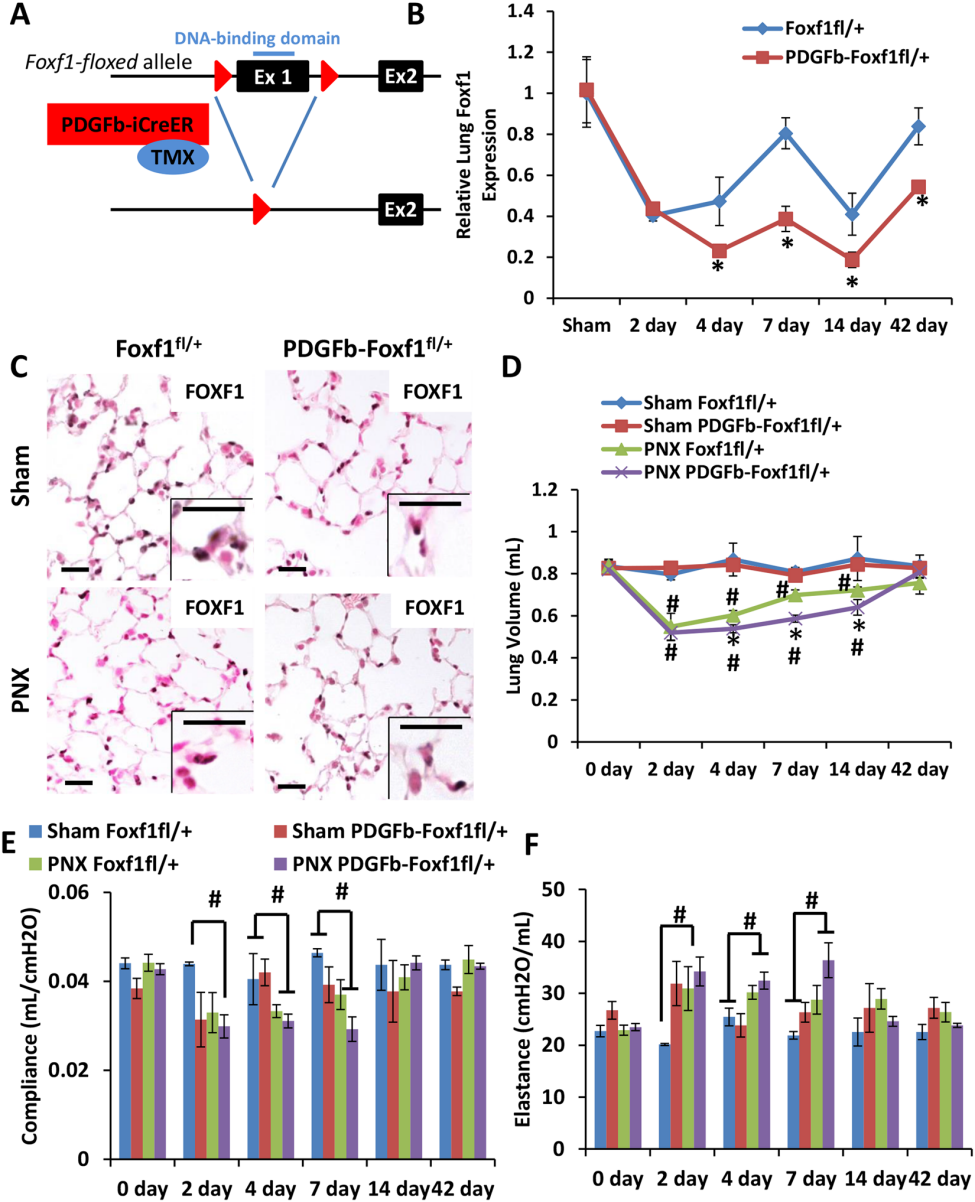
Delayed lung regeneration after PNX in mice with endothelial *Foxf1* deficiency. (**A**) Schematic representation of generation of *PDGFb-iCre/Foxf1* ^*fl*/+^ mice. LoxP sites were introduced to the *Foxf1* gene flanking Exon 1, which contains the DNA-binding domain. Mice possessing the *Foxf1-floxed* allele were bred to tamoxifen-inducible *PDGFb-iCreER* mice to generate mice heterozygous for the *Foxf1-floxed* allele in endothelial cells. (**B**) *Foxf1* mRNA was decreased following PNX. *PDGFb-iCre/Foxf1* ^*fl*/+^ mice had significantly less *Foxf1* mRNA following PNX than control mice. (**C**) FOXF1-staining was decreased in *PDGFb-iCre/Foxf1* ^*fl*/+^ mice following PNX. (**D**) PNX reduced lung volume by about 40% in control and *PDGFb-iCre/Foxf1* ^*fl*/+^ mice. Lung regeneration was delayed in *PDGFb-iCre/Foxf1* ^*fl*/+^ mice resulting in significantly smaller lungs from day 4 through 14. Lung regeneration was complete by 42 days post-PNX. (**E**,**F**) Compliance and elastance were altered by PNX but did not differ between control and *PDGFb-iCre/Foxf1* ^*fl*/+^ mice. *p < 0.05 compared to control; #p < 0.05 compared to sham. Scale bars are 20 μm.

Mice carrying the *Foxf1-floxed* allele, in which LoxP sites flank the first exon of the *Foxf1* gene encoding the DNA binding domain, have been described previously^10, 11^. *Foxf1-floxed* mice were bred with *PDGFb-iCre* mice, a tamoxifen inducible *Cre* that targets endothelial cells^11, 15^. Mice heterozygous for the *Foxf1-floxed* allele (*PDGFb-iCre/Foxf1* ^*fl*/+^, abbreviated *PDGFb-Foxf1* ^*fl*/+^) were utilized in this study (Fig. 1A). No differences were observed between *Foxf1* ^*fl*/+^ and *PDGFb-iCre/Foxf1* ^*fl*/+^ mice in the absence of tamoxifen, nor did tamoxifen administration affect *Foxf1* ^*fl*/+^ mice lacking *Cre*.

### Partial pneumonectomy (PNX) surgery

Pneumonectomy surgery was performed as previously described^16^. Briefly, under isoflurane anesthesia, 8–12 week old control or *PDGFb-iCre/Foxf1* ^*fl*/+^ mice were intubated to maintain pulmonary pressure. The left lung lobe was exposed via surgical incision into the intercostal space. The left bronchiole and supporting vasculature were ligated by surgical clamp, the left lung lobe was excised, and the thoracic cavity closed with suture. Negative pressure was returned to the thoracic cavity following wound closure by drawing air out using a 3 cc syringe with needle placed into the thoracic cavity prior to closure. Sham operated mice were treated the same as surgical mice without removal of the lung lobe, they still were intubated and the thoracic cavity breached via incision into the intercostal space. Following surgery mice were given Buprenex for pain management and monitored for adverse side effects. Tamoxifen (3 mg/day, Sigma) was administered by oral gavage the day prior to and the morning of PNX surgery.

### Evaluation of lung function, real-time qRT-PCR, Western blot, and immunostaining

Evaluation of lung volume and function was performed on days 2, 4, 7, 14, and 42 post-PNX. The middle right lobe was collected for RNA and protein, while the remaining right lobes, cranial, caudal and accessory, were imbedded for immunostaining. Alterations in lung volume and function following PNX were assessed using the Flexivent small animal ventilator (SCIREQ) as previously described^17^. Protein extracts were used for either Western blot analysis as previously described^18–20^ or gel zymography. RNA was isolated according to manufacturer’s protocol and reverse transcribed using Applied Biosystems high capacity reverse transcription kit according to manufacturer’s protocol as previously described^20–23^. Analysis of changes in gene expression was performed using a StepONE qRT-PCR machine (Applied Biosystems) and inventoried Taqman probes (Applied Biosystems, Supplemental Table 1) as previously described^20–23^. Lungs were gravity inflated with 4% paraformaldehyde prior to paraffin imbedding. Immunohistochemistry and immunofluorescence were performed on 5 μm paraffin sections and stained as previously described^20–22, 24–28^. Antibodies used in this study were FOXF1 (ref. 10), Ki-67 (1:200 (immunohistochemistry, IHC), 1:200 (immunofluorescence, IF);Thermo Scientific), PCNA (1:2000 (IHC), 1:500 (IF); Roche Diagnostics), PH3 (1:1000; Santa Cruz Biotechnology), TIMP3 (1:750;Santa Cruz Biotech), SOX17 (1:250; 7 Hills Bioreagents), SPC (1:500; 7 Hills Bioreagents), PECAM-1 (1:1000 (WB); Abcam), ENDOMUCIN (1:100; Abcam), BrdU (1:500 (IF); Iowa Hybridoma Bank), Cleaved Caspase 3 (1:1000; R&D Systems), CDKN1A (P21^Waf1/Cip1^)(1:1000; Cell Signaling Technolgy), CDKN2B (P15^INK4b^)(1:1000; Aviva Systems Biology), MMP14 (1:200; Abcam), β-ACTIN (1:6000; Santa Cruz Biotechnology). Images were collected using a Zeiss Axioplan 2 microscope and AxioVision software.

### ChIPseq and RNAseq

Formaldehyde cross-linked chromatin was sonicated using Covaris S220 to 200–300 bp and ChIP was performed using SX-8G IP-STAR robot (Diagenode) using the AF4798 antibody. ChIP-Seq library was prepared using ChIPmentation procedure and libraries were sequenced using Illumina HiSeq. 2500 at CCHMC sequencing core. Data analysis was performed using the BioWardrobe platform^29^. Briefly, ChIP-Seq reads were aligned by Bowtie to the mouse genome (mm10); only unique reads with no more than one mismatch were kept. Reads were extended to estimated fragment length, normalized to total mapped read number and displayed as coverage on a mirror of the University of California Santa Cruz (UCSC) genome browser. MACS2^30^ was used to identify islands of enrichment. FOXF1 sequence logos were identified with MEME-ChIP^31^. FOXF1 ChIPseq data was aligned with previously published ChIPseq data for histone methylation (GEO Accession GSE31039) using the BioWardrobe platform.

RNAseq analysis was performed on FACS-sorted endothelial cells (CD45^−^CD31^+^CD326^−^) from control and *PDGFb-iCre/Foxf1* ^*fl*/+^ lungs 4 days after PNX surgery. Changes in gene expression were determined using DESeq and analyzed as previously described^11^. Heat map was generated from differentially expressed genes using JMP Genomics 6.0.

ChIPseq and RNAseq data are available as GEO accession GSE100149.

### Analysis of lung air space

Changes in lung morphology were evaluated using the mean intercept method of evaluating respiratory versus air space within the lung. H&E stained 20x fields of lungs from sham operated and PNX mice during the first 2 weeks of recovery were analyzed. Analysis was performed by placing a lattice over the image and counting whether intersections landed on respiratory space or air space. Data is represented as percent of air space. 15 random fields from 4 mice per group were analyzed.

### Analysis of ENDOMUCIN staining

Changes in endothelial cell coverage in the lung were evaluated by measuring fluorescence using ImageJ software. ENDOMUCIN-stained 20x fields of lungs from sham operated and PNX mice at four and seven days of recovery were analyzed. Analysis was performed by measuring fluorescent intensity. Data is represented as mean fluorescent intensity. 15 random fields from 4 mice per group were analyzed.

### Fluorescence assisted cell sorting (FACS)

Isolation of endothelial and epithelial cell populations by FACS was performed as previously described^10, 11, 32, 33^. Hematopoetic cells were eliminated based on CD45 expression. Endothelial cells were then defined from CD45^−^ cells as having high CD31 expression and lacking CD326 expression (CD45^−^CD31^+^CD326^−^). Epithelial cells were defined from CD45^−^ cells as having high CD326 expression and lacking CD31 expression (CD45^−^CD31^−^CD326^+^). Antibodies used were CD45 (eBioscience, clone 30-F11), CD31 (eBioscience, clone 390), and CD326 (eBioscience, clone G8.8). Cells were separated using Five-laser FACSAria II, BD Biosciences. Purified cells were used for qRT-PCR and RNAseq.

FACS isolated endothelial cells (CD45^−^CD31^+^CD326^−^) were analyzed for DNA content using DRAQ5, a cell permeable dye. Percentages of cells in G_0_/G_1_ and S/G_2_/M phases of the cell cycle were measured. 3 mice were used per group.

### Zymography

Zymography was performed on whole lung protein samples collected on day 7 post-PNX. Samples were homogenized under non-reducing conditions and protein concentration determined by Applied Biosystems DC protein assay according to manufacturer’s instructions. Ten μg of total protein were loaded per sample and run on zymography gel according to manufacturer’s protocol (10% gelatin gel, NOVEX). Gel images were captured on a Canon scanner (Canoscan LiDE 90) and band intensity determined by ImageJ software.

### Statistical analysis

Statistical analysis was performed using Excel software. Student’s t-test analysis was used to determine significance which was set at p ≤ 0.05. All data are represented as mean ± standard error of mean (SEM).

## Results

### Foxf1 deficiency in endothelial cells delays lung regeneration after partial pneumonectomy

To determine the role of FOXF1 in endothelial cells during lung regeneration, we performed partial pneumonectomy (PNX) surgery on mice with endothelial-specific *Foxf1* deficiency. Since deletion of both *Foxf1* alleles from endothelial cells is lethal^11^, mice containing a deletion of one *Foxf1* allele (*PDGFb-iCre/Foxf1* ^*fl*/+^) were used in the present study (Fig. 1A). Tamoxifen was administered prior to PNX surgery to activate *Cre*. After PNX, *Foxf1* mRNA expression was significantly decreased in both *PDGFb-iCre/Foxf1* ^*fl*/+^ and control mice (Fig. 1B), *Foxf1* mRNA levels were significantly lower in *PDGFb-iCre/Foxf1* ^*fl*/+^ mice compared to control mice as determined by qRT-PCR analysis (Fig. 1B). Consistent with decreased *Foxf1* mRNA, FOXF1 staining was decreased in *PDGFb-iCre/Foxf1* ^*fl*/+^ lungs compared to controls (Fig. 1C). Evaluation of FOXF1 distribution in sham-operated and regenerating lungs showed that FOXF1 is widely distributed in the alveolar region but can only be detected in a subset of endothelial cells within pulmonary arteries and veins (Supplemental Fig. 1). Measurement of lung function showed PNX caused a 40% decrease in lung volume in control and *PDGFb-iCre/Foxf1* ^*fl*/+^ mice 2 days after surgery, consistent with removal of the left lung lobe. Lung volume increased in mice from both genotypes from day 2 following PNX, ultimately returning to sham levels by 42 days (Fig. 1D). Regeneration was delayed in *PDGFb-iCre/Foxf1* ^*fl*/+^ mice compared to controls, as shown by significantly smaller lung volumes in these mice from days 4 through 14 (Fig. 1D). Interestingly, despite delayed regeneration *Foxf1*-deficient lungs had normal size of alveoli (Supplemental Fig. 2A,B) and lung function was not affected as shown by measurements of lung compliance, elastance, and resistance (Fig. 1E,F, Supplemental Fig. 2C). Consistent with these findings lung collagen content and distribution was unaltered as shown by qRT-PCR analysis of *Col1a1* and *Col3a1* (Supplemental Fig. 2E) as well as Masson’s trichrome stain (Supplemental Fig. 2F). Tamoxifen administration did not affect lung regeneration as *Foxf1* ^*fl*/+^ and *PDGFb-iCre/Foxf1* ^*fl*/+^ mice in the absence of tamoxifen showed normal kinetics of lung volume recovery (Supplemental Fig. 2D). Thus, *Foxf1* expression in endothelial cells promotes lung regeneration following PNX.

### ChIPseq analysis identifies FOXF1 target genes in endothelial cells

**Figure 2:**
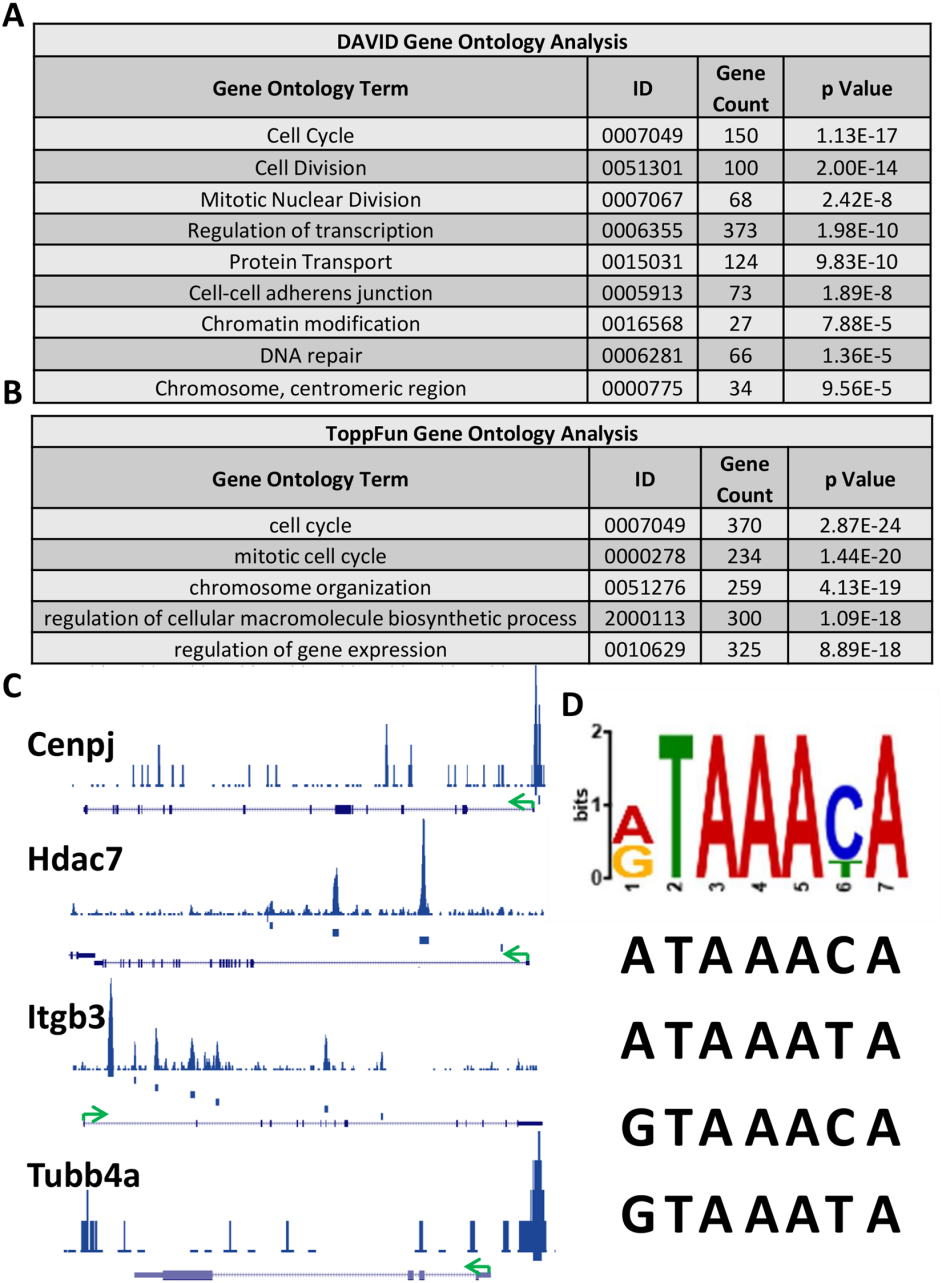
Identification of FOXF1 target genes in endothelial cells by ChIPseq. Gene ontology analysis using (**A**) DAVID or (**B**) ToppFun performed on ChIPseq data shows top biological processes associated with FOXF1 chromatin binding. Ontology identification numbers, number of genes from the list of changed genes per term, and p value are listed for each classification. (**C**) Schematic of FOXF1 binding in *Cenpj*, *Hdac7*, *Itgb3*, and *Tubb4a* DNA regulatory regions as determined by ChIPseq. (**D**) FOXF1 consensus binding sequence and specific DNA sequences as determined by ChIPseq.

To determine molecular mechanisms whereby FOXF1 stimulates lung regeneration, ChIPseq analysis was performed in the MFLM-91U pulmonary endothelial cell line. ChIPSeq data analysis identified 16024 FOXF1 binding sites. MEME-ChIP^31^ analysis of the sequence under the peaks identified a TAAACA motif (Fig. 2D) which is similar to the motif previously reported for FOXF1^34^ (Fig. 2D), 8916 genes were located in the vicinity of FOXF1 binding sites. Of these genes, FOXF1 bound to known promoter regions for 2196 genes. Gene ontology analysis performed using DAVID^35^ and ToppFun^36^ softwares showed that cell cycle regulating genes were the major targets of FOXF1 transcription factor binding (Fig. 2A,B). The main biological processes affected by FOXF1 target genes included cell cycle regulation, cell division, mitotic cell division, regulation of transcription, chromosome organization, and DNA repair (Fig. 2A,B). Other gene categories associated with FOXF1 binding include: transcription cofactor activity, transcription factor binding, and regulation of gene expression. ChIPseq demonstrated that FOXF1 bound to chromatin at *Flt1*, *Kdr*, *Notch2*, *Itgb3*, *Pdgfb*, *Pecam1*, *S1pr1*, and *Tek* gene loci, all of which have been previously shown to be direct FOXF1 target genes^10, 11, 37^ (Supplemental Table 2). Among the novel FOXF1 targets identified were centromere protein J (*Cenpj*) and tubulin beta-4A (*Tubb4a*), both of which regulate mitotic cell division, mitotic spindle formation, and chromatin separation^38–40^ (Supplemental Table 2, Fig. 2C). Histone deacetylase (*Hdac*) 5 and 7 gene loci were also bound by FOXF1 (Supplemental Table 2, Fig. 2C, Supplemental Fig. 3). HDAC proteins regulate histone tertiary structure which is important in both gene transcription and cell division^41^. FOXF1 also bound to promoter regions of *Cdkn1a*, *Cdkn2b*, *Ccnd3*, and *Cd44*, genes critical for endothelial proliferation^42, 43^ (Supplemental Table 2, Supplemental Fig. 3). Other FOXF1 target genes identified by ChIPseq include: *Adamts9*, *Itgb4*, *Ptgs1*, *Ptgs2*, and *Spry4* (Supplemental Table 2, Supplemental Fig. 3).

### Identification of FOXF1 target genes in regenerating lungs

**Figure 3:**
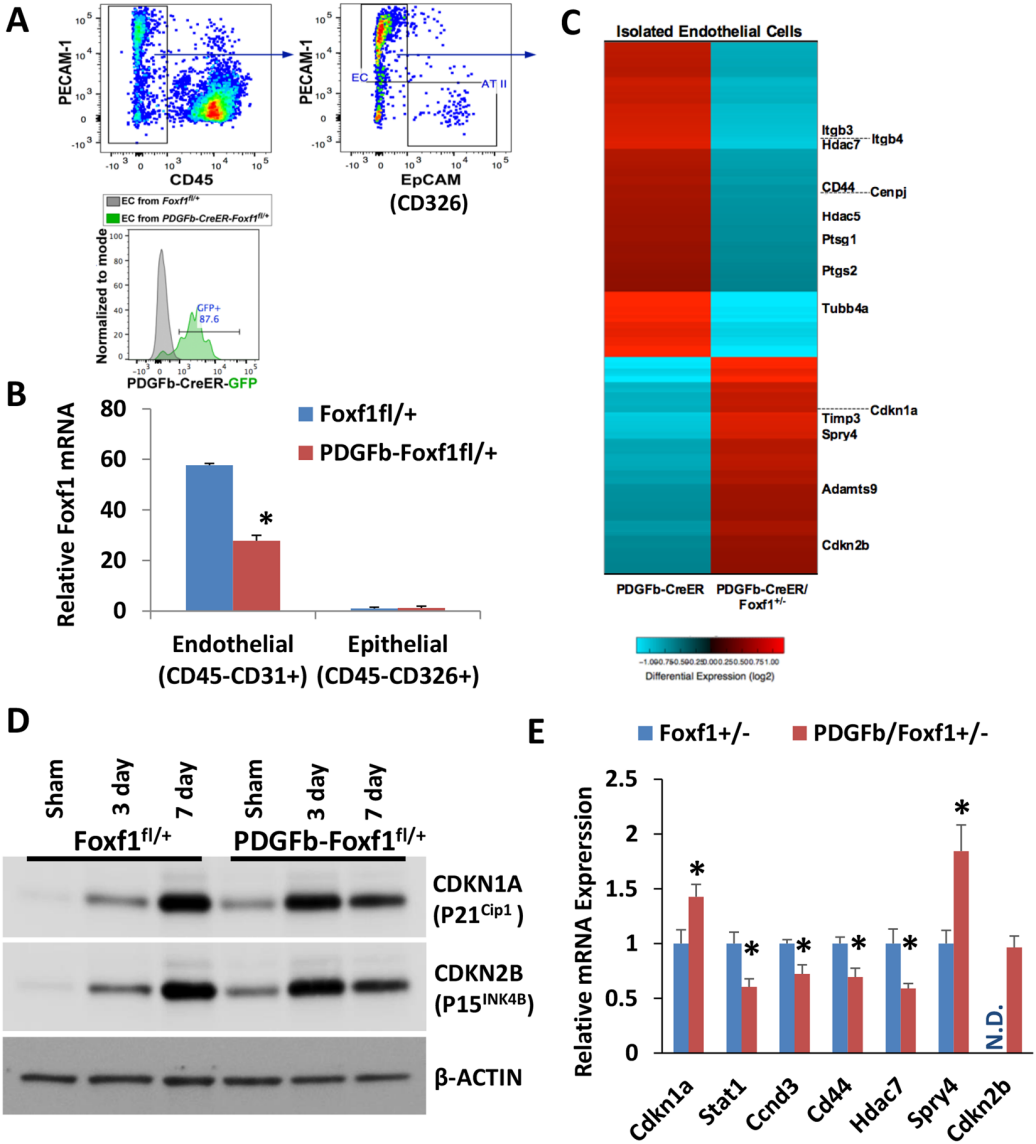
Identification of FOXF1 target genes in endothelial cells from regenerating lungs. (**A**) FACS-sorting strategy to isolate populations of endothelial (CD45^−^CD31^+^CD326^−^) and epithelial (CD45^−^CD31^−^CD326^+^) cells, showing GFP expression in *PDGFb-iCre* expressing mice. (**B**) *Foxf1* mRNA was highly expressed in endothelial cells but not in epithelial cells. Endothelial cells from *PDGFb-iCre/Foxf1* ^*fl*/+^ mice had significantly less *Foxf1* mRNA than endothelial cells from control mice. (**C**) Heat map showing significant changes in expression of 1047 genes in endothelial cells from *Foxf1* ^*fl*/+^ and *PDGFb-iCre/Foxf1* ^*fl*/+^ lungs after PNX. (**D**) Western blot analysis showed increased protein levels for CDKN1A (P21^Cip1^) and CDKN2B (P15^Ink4b^) in *PDGFb-iCre/Foxf1* ^*fl*/+^ lungs in sham mice and 3 days after PNX. Cropped gels are presented here with full gel available in Supplemental Fig. 8. (**E**) qRT-PCR analysis showed significant changes in mRNA expression of several FOXF1 target genes in endothelial cells from *PDGFb-iCre/Foxf1* ^*fl*/+^ lungs. *Cdkn2b* was not detectable (N.D.) in control samples. *p < 0.05 compared to control.

Next we determined whether *Foxf1*-deficiency changed expression of these genes *in vivo*. FACS was used to purify populations of endothelial (CD45^−^CD31^+^CD326^−^) and epithelial (CD45^−^CD31^−^CD326^+^) cells from regenerating lungs (Fig. 3A). Purity of populations isolated by FACS was confirmed by the presence of *Pecam1* in endothelial cells and *Sftpc* in epithelial cells as determined by qRT-PCR (Supplemental Fig. 4A,B). *Foxf1* was expressed in endothelial cells but not epithelial cells (Fig. 3B), which is consistent with previous studies^10, 11^. A 50% decrease in *Foxf1* mRNA was found in *PDGFb-iCre/Foxf1* ^*fl*/+^ endothelial cells compared to endothelial cells from control mice (Fig. 3B), consistent with deletion of one *Foxf1* allele. To identify transcripts regulated by FOXF1, FACS-sorted endothelial cells were analyzed by genome-wide RNAseq analysis. Significant changes in expression for over a thousand genes were found in endothelial cells from *PDGFb-iCre/Foxf1* ^*fl*/+^ lungs 4 days after PNX (Fig. 3C). Consistent with ChIPseq data (Fig. 2A,B), mRNA expression of multiple cell cycle regulators was altered. These genes include: *Cenpj*, *Tubb4a*, *Adamts9*, *Hdac5*, *Hdac7*, *Itgb3*, *Itgb4*, *Ptgs1*, *Ptgs2*, and *Spry4* (Table 1). *Cd44*, which promotes endothelial cell proliferation, was one of the genes with decreased expression in *Foxf1*-deficient endothelial cells (Table 1). Among the genes with increased mRNA levels in *PDGFb-iCre/Foxf1* ^*fl*/+^ mice were the cell cycle inhibitory proteins *Cdkn1a*, *Cdkn2b*, and *Spry4* (Table 1) all of which inhibit cellular proliferation *in vitro* and *in vivo* ^44^. Expression of *Timp3* and *Adamts9*, both critical for ECM remodeling, was also increased in *PDGFb-iCre/Foxf1* ^*fl*/+^ endothelial cells (Table 1). qRT-PCR confirmed altered expression of these genes in *Foxf1*-deficient endothelial cells (Fig. 3E). qRT-PCR analysis also showed decreased expression of *Ccnd3* in *Foxf1*-deficient endothelial cells, supporting decreased cell cycle progression after PNX. Consistent with mRNA changes, Western blot analysis showed that protein levels of CDKN1A (P21^Cip1^) and CDKN2B (P15^Ink4b^) were increased in lungs from *PDGFb-iCre/Foxf1* ^*fl*/+^ mice at base line and 3 days after PNX (Fig. 3D), consistent with decreased proliferation at these time points. Levels of CDKN1A and CDKN2B increase in control lungs 7 days after PNX as proliferation starts to wane. Thus FOXF1 regulates multiple endothelial genes critical for lung regeneration, ECM remodeling, and cell cycle progression.

**Table 1:** Proliferation-related genes altered in endothelial cells from *PDGFb-Foxf1* ^*fl*/+^ mice 3 days after PNX.

**Functional Class**	**Gene**	**Refseq**	**Foxf1** ^**fl**/+^ **FKPM**	**PDGFb-Foxf1** ^**fl**/+^	**Fold Δ**
Cell cycle regulators	*Cdkn1a*	NM_001111099	1.5101	3.2366	1.965586
	*Cdkn2b*	NM_007670	5.70687	7.08432	1.138437
Cytokinesis	*Cenpj*	NM_001014996	3.57699	2.17372	0.557307
	*Tubb4a*	NM_009451	2.29284	0.0390402	0.015615
Proliferation related genes	*Adamts9*	NM_175314	3.66515	5.39029	1.348743
	*Cd44*	NM_001039151	5.53783	3.06149	0.506993
	*Hdac5*	NM_001077696	6.077	3.47019	0.523688
	*Hdac7*	NM_001204276	12.2208	5.48882	0.411897
	*Itgb3*	NM_016780	3.64954	1.93063	0.485143
	*Itgb4*	NM_133663	2.51632	0.911884	0.33234
	*Ptgs1*	NM_008969	27.3028	12.3782	0.415776
	*Ptgs2*	NM_011198	3.22424	1.22177	0.347513
	*Spry4*	NM_011898	14.5635	26.4423	1.665108
	*Timp3*	NM_011595	141.985	246.456	1.591863

### Decreased proliferation in PDGFb-iCre/Foxf1^fl^^/+^ mice after PNX

**Figure 4:**
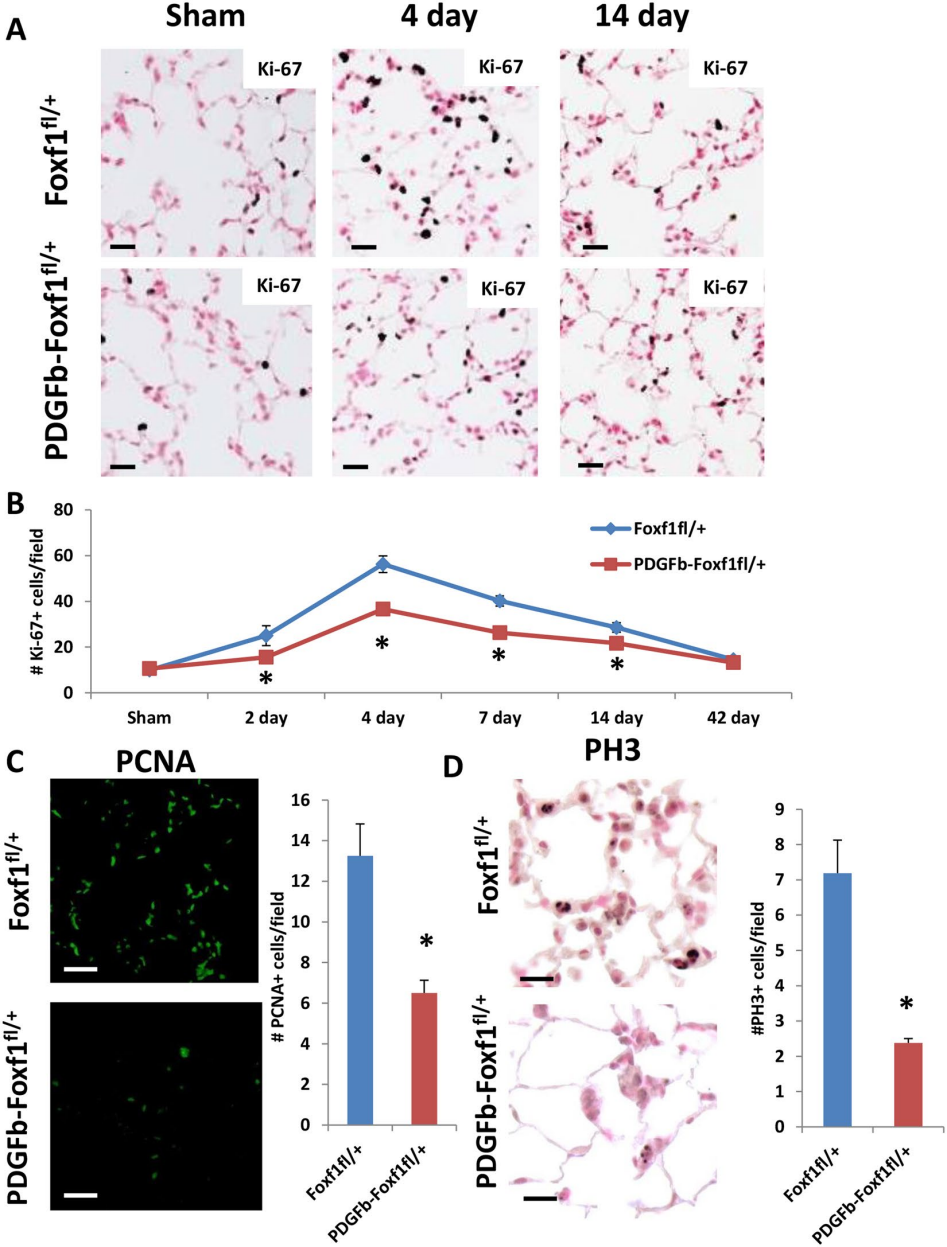
Decreased proliferation in *PDGFb-iCre/Foxf1* ^*fl*/+^ mice after PNX. (**A**,**B**) Ki-67 staining showed increased proliferation following PNX. The number of Ki-67 positive cells was decreased in *PDGFb-iCre/Foxf1* ^*fl*/+^ mice compared to control following PNX. Ki-67 positive cells were counted in 15 random microscope fields (n = 4 mice per group). (**C**) PCNA staining showed decreased proliferation in *PDGFb-iCre/Foxf1* ^*fl*/+^ mice compared to controls. (**D**) PH3 staining showed decreased proliferation in *PDGFb-iCre/Foxf1* ^*fl*/+^ mice compared to controls. *p < 0.05 compared to control. Scale bars are 20 μm.

**Figure 5:**
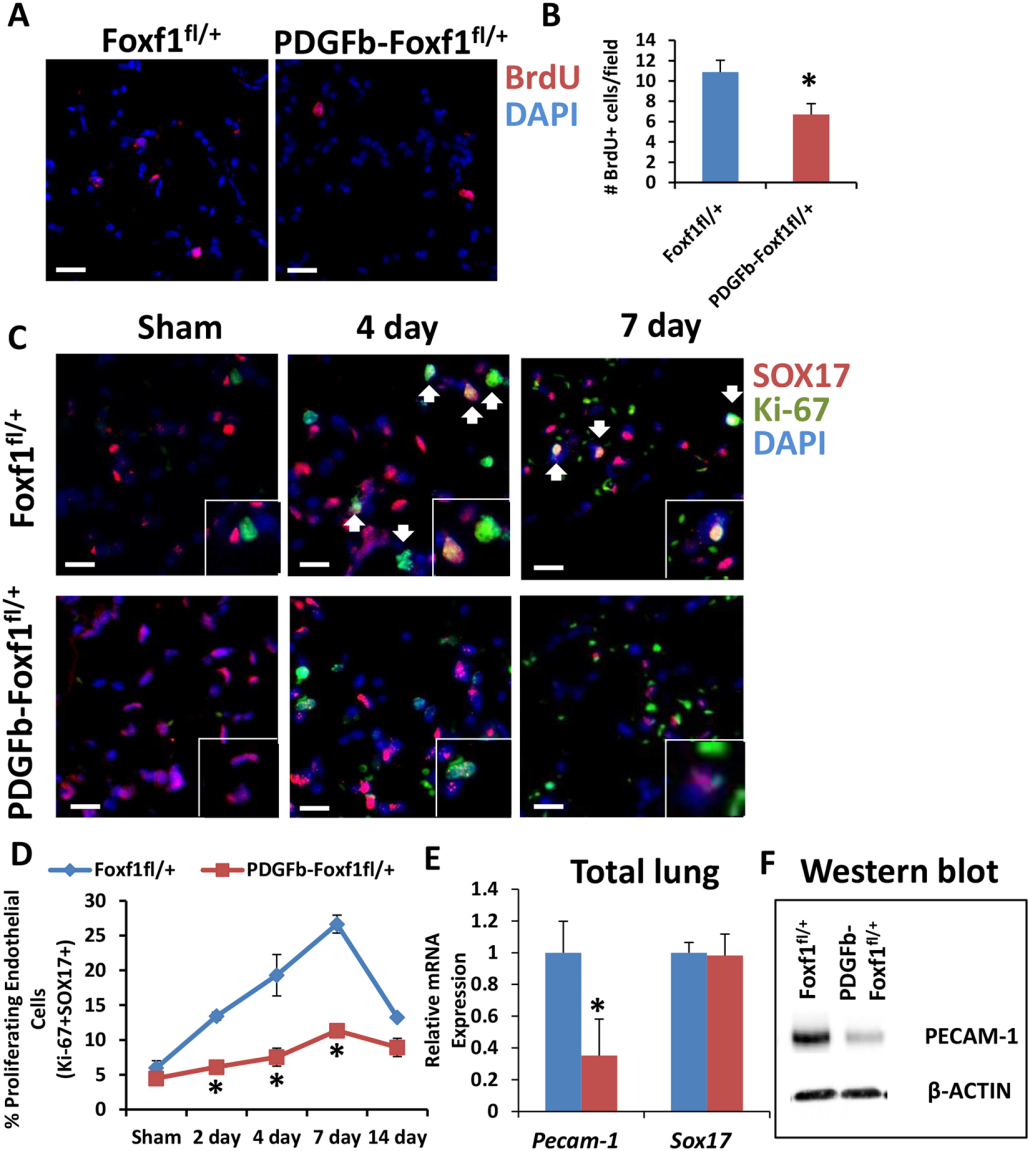
Decreased endothelial proliferation in *PDGFb-iCre/Foxf1* ^*fl*/+^ mice after PNX. (**A**,**B**) BrdU incorporation showed less proliferation in lungs from *PDGFb-iCre/Foxf1* ^*fl*/+^ mice than control following PNX. (**C**,**D**) Co-immunofluorescence experiments with SOX17 to mark endothelial cells and Ki-67 to mark proliferating cells showed that endothelial cell proliferation increased following PNX. Endothelial proliferation was attenuated in *PDGFb-iCre/Foxf1* ^*fl*/+^ mice compared to controls. (**E**) qRT-PCR analysis showed decreased *Pecam-1* mRNA in lungs from *PDGFb-iCre/Foxf1* ^*fl*/+^ mice compared to controls, *Sox17* mRNA expression was unaltered. (**F**) Western blot analysis demonstrated decreased PECAM-1 protein in *PDGFb-iCre/Foxf1* ^*fl*/+^ mice following PNX compared to controls. Cropped gels are presented here with full gel available in Supplemental Fig. 8. *p < 0.05 compared to control. Scale bars are 20 μm.

Since FOXF1 binds to and regulates expression of cell cycle regulatory genes, we examined cellular proliferation in *Foxf1*-deficient lungs in a PNX model of lung regeneration. Consistent with previous reports^3, 4^ we observed increased proliferation after PNX in control mice. However, the number of Ki-67-positive cells was significantly lower in *PDGFb-iCre/Foxf1* ^*fl*/+^ mice compared to controls (Fig. 4A,B). In agreement with Ki-67 staining, immunostaining for proliferating cell nuclear antigen (PCNA) or phospho-Histone 3 (PH3) showed significantly less cell proliferation in *PDGFb-iCre/Foxf1* ^*fl*/+^ mice compared to control mice (Fig. 4C,D). Furthermore, the number of cells undergoing DNA replication after PNX was decreased in lungs of *PDGFb-iCre/Foxf1* ^*fl*/+^ mice as determined by BrdU incorporation (Fig. 5A,B). As *PDGFb-iCre* specifically targets endothelial cells, immunostaining for Ki-67 and SOX17, a marker of endothelial cells^45^, was performed to visualize endothelial cells undergoing the cell cycle. In agreement with previous studies^3^, PNX surgery increased the number of proliferating endothelial cells in both mouse lines (Fig. 5C,D). Endothelial cell proliferation was significantly decreased in regenerating lungs of *PDGFb-iCre/Foxf1* ^*fl*/+^ mice compared to control (Fig. 5C,D). Consistent with decreased endothelial cell proliferation, Pecam-1 mRNA and protein were reduced in *PDGFb-iCre/Foxf1* ^*fl*/+^ lungs as determined by qRT-PCR and Western blot, respectively (Fig. 5E,F). ENDOMUCIN staining and quantification (Supplemental Fig. 5A,B) showed similar expression and patterning during the regenerative phase of post-PNX growth, consistent with maintenance of lung morphology between control and *PDGFb-iCre/Foxf1* ^*fl*/+^ mice during lung regeneration. Although proliferation of alveolar type II epithelial cells also increased after PNX, it was largely unchanged between control and *PDGFb-iCre/Foxf1* ^*fl*/+^ lungs (Supplemental Fig. 5C,D). Staining for activated caspase 3 showed no difference between control and *PDGFb-iCre/Foxf1* ^*fl*/+^ lungs (Supplemental Fig. 6A), suggesting apoptosis does not contribute to delayed lung regeneration in *PDGFb-iCre/Foxf1* ^*fl*/+^ mice. DNA content was analyzed in FACS-sorted endothelial cells (CD45^−^CD31^+^CD326^−^) by DRAQ5 at 19 days post-PNX to evaluate endothelial proliferation during the late phase of regeneration. This analysis showed that a small number of endothelial cells still proliferate at this late stage of regeneration, with decreased numbers of endothelial cells undergoing S, G_2_, and M phases of the cell cycle in *PDGFb-iCre/Foxf1* ^*fl*/+^ lungs (Supplemental Fig. 6B,C). Therefore, deletion of one *Foxf1* allele from endothelial cells was sufficient to delay endothelial proliferation and lung regeneration after PNX.

**Figure 6:**
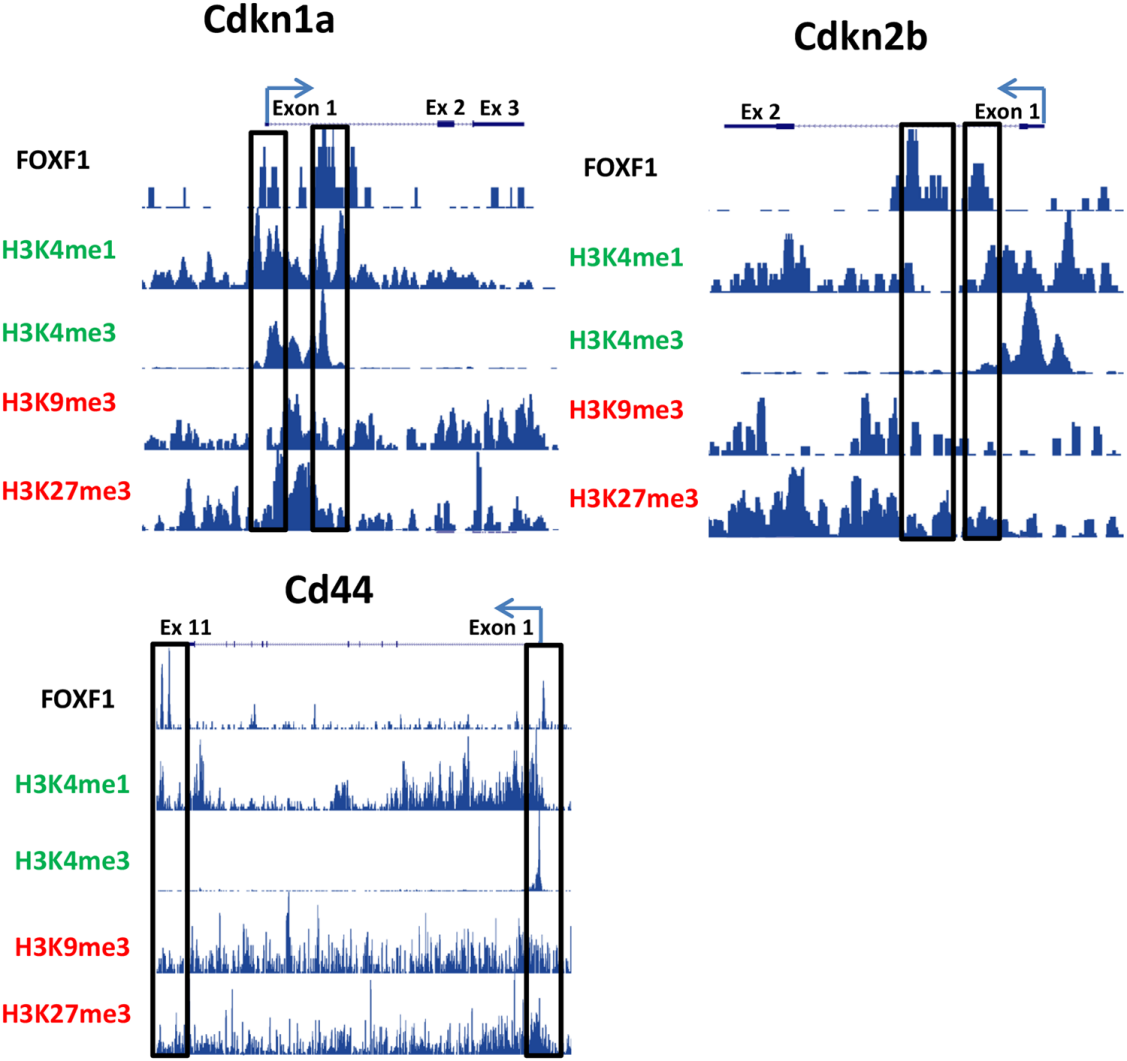
ChIPseq shows FOXF1 directly regulates genes critical for cell cycle progression. Chipseq analysis performed for FOXF1 was aligned with published Chipseqs for histone modifications favoring gene transcription (H3K4me1, H3K4me3) or repression (H3K9me3, H3K27me3). *Cdkn1a* (P21^Cip1^) and *Cdkn2b* (P15^INK4b^) genes had multiple FOXF1 binding sites near the promoter region and with the introns. Cd44 had a single FOXF1 binding site near the transcriptional start sites as well as near the end of the transcript. Areas of significant FOXF1 binding to chromatin are marked by black boxes. Direction of gene transcription and transcriptional start sites are marked with an arrow.

Since analysis of FOXF1 binding to chromatin indicated cell cycle regulation is the main gene ontology signature for FOXF1, we further investigated three genes critical for endothelial proliferation, *Cdkn1a*, *Cdkn2b*, and *Cd44*. These genes were analyzed to determine whether their respective FOXF1 binding regions had positive or negative histone methylation marks as determined by ChIPseq. FOXF1 bound to *Cd44* in the promoter region (−494/−1014 bp) and within the final exon (+6058/+5857 bp) (Fig. 6, Supplemental Table 2). FOXF1 binding regions of *Cd44* had tri- and mono-methylation of histone 3 lysine 4 (H3K4me1 and H3K4me3), both of which promote transcriptional activation^46–49^ (Fig. 6), suggesting FOXF1 stimulates *Cd44* transcription. These results are consistent with decreased *Cd44* mRNA levels in *Foxf1*-deficient endothelial cells (Fig. 3E). In contrast, FOXF1 binding regions for *Cdkn1a* (−151/+394 bp and +1447/+1627 bp) and *Cdkn2b* (+897/+1073 bp and +1866/+2032 bp) often aligned with H3K4me1 and H3K4me3 but were also positive for H3K27 tri-methylation (H3K27me3) which causes transcriptional silencing^50^ (Fig. 6). Consistent with the presence of H3K27me3 gene silencing marks, mRNA and protein for *Cdkn1a* (P21^Cip1^) and *Cdkn2b* (P15^Ink4b^) were increased in *Foxf1*-deficient lungs (Fig. 3D,E). Thus, FOXF1 binds to both enhancer and repressor regions to differentially regulate gene expression in regenerating endothelial cells.

### Increased TIMP3 expression, decreased MMP14 activity in PDGFb-iCre/Foxf1^fl^^/+^ lungs

**Figure 7:**
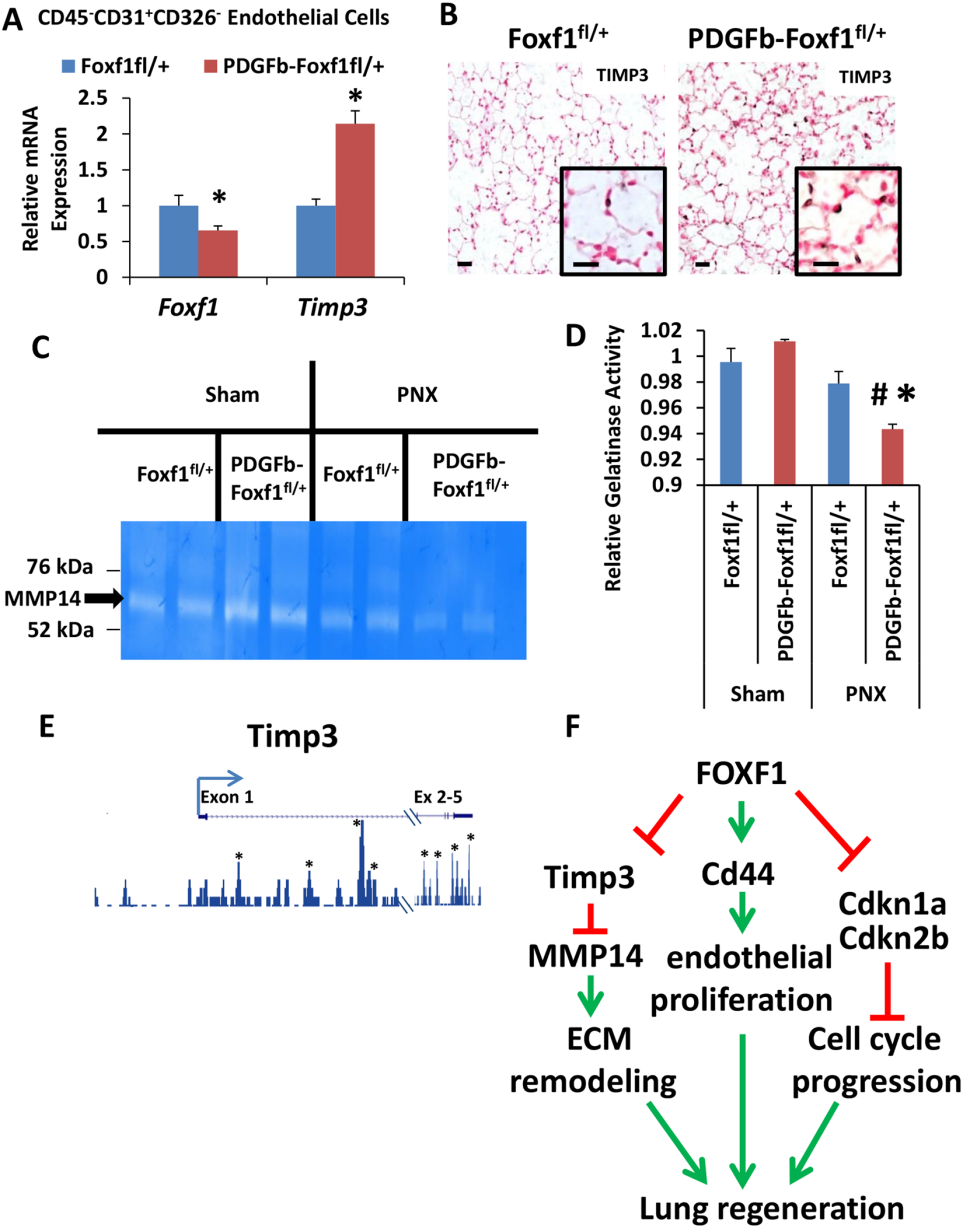
Decreased MMP14 activity in *PDGFb-iCre/Foxf1* ^*fl*/+^ lungs after PNX. (**A**) FACS-sorted endothelial cells (CD45^−^CD31^+^CD326^−^) showed decreased *Foxf1* but increased *Timp3* mRNAs in *PDGFb-iCre/Foxf1* ^*fl*/+^ lungs compared to controls. (**B**) TIMP3 staining was increased in *PDGFb-iCre/Foxf1* ^*fl*/+^ mice after PNX. (**C**) Representative zymography gel showing MMP14 gelatinase activity in lung samples collected after sham or PNX surgery in control and *PDGFb-iCre/Foxf1* ^*fl*/+^ mice. A cropped gel is presented here with full gel available in Supplemental Fig. 9. (**D**) Quantification of zymography gels shows decreased MMP14 activity in *PDGFb-iCre/Foxf1* ^*fl*/+^ lungs compared to controls. (**E**) ChIPseq analysis showing multiple FOXF1 binding sites within *Timp3* gene locus. (**F**) Diagram showing multiple pathways whereby FOXF1 induces lung regeneration after PNX. *p < 0.05 compared to control; ^#^p < 0.05 compared to sham. Scale bars are 20 μm.

Rearrangement of the extracellular matrix (ECM) by MMPs is a major component of alveologenesis and it was previously shown that MMP14 induces lung regeneration after PNX^3^. While neither Mmp14 mRNA nor protein was altered (Supplemental Fig. 7), mRNA expression of *Timp3*, an inhibitor of MMP14^51^, was increased in FACS-sorted *PDGFb-iCre/Foxf1* ^*fl*/+^ lung endothelial cells as shown by RNAseq (Fig. 3C) and qRT-PCR (Fig. 7A). FOXF1 directly bound to *Timp3* DNA regulatory regions located in the promoter and first intron (Fig. 7E and Supplemental Table 2), indicating that *Timp3* is a direct transcriptional target of FOXF1. Consistent with this hypothesis, TIMP3 immunostaining was increased and more TIMP3-positive cells were observed in regenerating lungs from *PDGFb-iCre/Foxf1* ^*fl*/+^ mice compared to controls (Fig. 7B). Furthermore, zymography was used to demonstrate that gelatinase activity of MMP14, a functional readout of TIMP3 activation^51^, was significantly decreased in regenerating lungs from *PDGFb-iCre/Foxf1* ^*fl*/+^ mice (Fig. 7C,D). Thus, reduced MMP14 activation can contribute to delayed lung regeneration in *PDGFb-iCre/Foxf1* ^*fl*/+^ mice. Altogether, FOXF1 stimulates lung regeneration by regulating transcription of endothelial genes critical for ECM remodeling and cell cycle progression (Fig. 7F).

## Discussion

Lung injury or lung reduction surgery diminishes the capacity for gas exchange within the lung, causing hypoxia within the tissues of the body which can lead to organ failure. Due to the profound need for efficient lung function, pathways through which lung regeneration can be expedited could dramatically help overall survival. In this study, we found that FOXF1, a transcription factor expressed in endothelial cells, is critical for lung regeneration following PNX. Mice with endothelial-specific deletion of one *Foxf1* allele have slower rates of lung regeneration compared to control mice. Utilizing a combination of RNAseq and ChIPseq analyses novel transcriptional targets of FOXF1 where identified in this study. Our data suggests FOXF1 stimulates lung regeneration by regulating endothelial genes critical for ECM remodeling (*Timp3*, *Adamts9*), cell cycle regulation (*Cdkn1a*, *Cdkn2b*), and cytokinesis (*Tubb4a*, *Cenpj*, *Hdac5*, and *Hdac7*). DNA regulatory regions of these genes possess high-affinity binding sites for FOX proteins that were occupied by FOXF1 bound to chromatin. The functional importance of these FOXF1-binding regions was supported by epigenetic marks such as single and triple methylation of lysine 4 of histone 3 which mark enhancer regions^46–49^ as well as triple methylation of lysine 27 of histone 3 which marks regions of gene repression^50^. Thus, FOXF1 is an important regulator of lung regeneration after PNX.

Our data are consistent with published studies demonstrating that mice haploinsufficient for the global *Foxf1-null* mutation (*Foxf1* ^+/*−*^) exhibited decreased rates of liver regeneration following carbon tertrachloride administration^14^ and increased susceptibility to butylated hydroxytoluene (BHT)-induced lung injury^13^. Interestingly, *Foxf1* ^+/−^ mice have a variety of developmental defects including fusion of lung lobes and vessels^7, 52, 53^ as well as esophageal^7^ and gastrointestinal abnormalities^54^. Therefore, these mice are not practical for use in PNX regeneration studies. Herein, we utilized an inducible mouse model to delete one *Foxf1* allele in an endothelial restricted manner in adult mice. This model enabled discovery of novel functions of FOXF1 as a transcriptional driver of lung regeneration. *PDGFb-iCre/Foxf1* ^*fl*/+^ mice had slower rates of regeneration up to two weeks after PNX, but *Foxf1*-deficient lungs did ultimately regenerate back to pre-PNX volumes by 6 weeks. Nineteen days after PNX, a small percentage of endothelial cells were still proliferating in control and *PDGFb-iCre/Foxf1* ^*fl*/+^ lungs. This low level of proliferation likely continues in *PDGFb-iCre/Foxf1* ^*fl*/+^ mice until the stresses that stimulate lung regeneration are quenched, which occurs quicker in control lungs where endothelial cells proliferate faster. Previous research has shown that pulmonary microvascular endothelial cells stimulate lung regeneration via VEGFR2 and FGFR1^3^; however, expression of these receptors was not altered in the current study, suggesting they do not play a role in decreased endothelial cell proliferation in *PDGFb-iCre/Foxf1* ^*fl*/+^ lungs. It was further shown that MMP14 activity was essential for post-PNX growth as administration of anti-MMP14 monoclonal antibodies blocked lung regeneration^3^. While *Mmp14* expression was not altered in the current study, regenerating *PDGFb-iCre/Foxf1* ^*fl*/+^ lungs had increased mRNA and protein for Timp3, an inhibitor of MMP14 activity. Consistent with increased TIMP3 immunostaining, MMP14 activity was decreased in *Foxf1*-deficient lungs, suggesting reduced MMP14 activity can contribute to delayed lung regeneration in *PDGFb-iCre/Foxf1* ^*fl*/+^ mice. Interestingly, mRNA expression of *Adamts9*, which regulates ECM remodeling similar to TIMP3^55, 56^, was also increased in *Foxf1*-deficient lungs. Since both *Adamts9* and *Timp3* are direct FOXF1 targets, FOXF1 may induce lung regeneration through transcriptional repression of these genes.

Previous studies have shown decreased cell proliferation in *Foxf1*-deficient embryos which resulted in decreased angiogenesis and smaller lung size^10^. *Foxf1* depletion in rhabdomyosarcoma cells increased levels of the CDK inhibitor *Cdkn1a* (P21^Cip1^), whereas *Foxf1*-overexpression reduced *Cdkn1a* levels^57^. ChIP analysis showed FOXF1 bound directly to the *Cdkn1a* promoter, contributing to its transcriptional repression^57^. Interestingly, in addition to the FOXF1 binding site identified in rhabdomyosarcoma cells by Milewski *et al*.^57^, the current study identified an additional high affinity FOXF1 binding region located within the first exon of the *Cdkn1a* gene. Since an endothelial cell line was used for ChIPseq in our studies, FOXF1 may regulate *Cdkn1a* through distinct regulatory regions in a tissue or cell-type specific manner. It is also possible that FOXF1 requires cofactors to efficiently bind chromatin and that expression of these cofactors in different cell-types could mediate the ability of FOXF1 to bind distinct DNA regulatory regions. The presence or absence of chromatin binding cofactors may determine whether FOXF1 functions as a transcriptional activator or repressor as both capacities were observed in this study. An important contribution of the current study is the use of a genome-wide approach to identify novel FOXF1 targets in endothelial cells. Analysis of ChIPseq data for which FOXF1 bound to the promoter regions showed a high predilection for genes affecting the cell cycle. Among these genes are positive and negative regulators of the cell cycle, genes essential for formation of the mitotic spindle and cytokinesis, and genes known to promote cell cycle induction. CYCLIN D3 promotes cell cycle progression via interaction with CDK4/6^58^. This study shows that FOXF1 binds *Cyclin D3* DNA regulatory regions and decreased expression could contribute to delayed lung regeneration in *PDGFb-iCre/Foxf1* ^*fl*/+^ mice. Our findings are consistent with increased expression of *Cdkn1a* and *Cdkn2b* (P15^Ink4b^) in *Foxf1-*deficient lungs, both of which inhibit cell cycle progression via the CDK2/4/6 pathway^59, 60^. Thus decreased FOXF1 in endothelial cells could result in decreased CDK activity, which in turn inhibits endothelial proliferation and delays lung regeneration after PNX.

In summary, this study shows FOXF1 induces lung regeneration via transcriptional regulation of endothelial genes critical for ECM remodeling, cell cycle progression, and cytokinesis. Therefore, small molecule compounds capable of promoting *Foxf1* transcription or protecting FOXF1 protein from degradation could provide a useful therapeutic for a variety of pulmonary diseases associated with delayed lung regeneration and repair.

## Electronic supplementary material

All supplemental files
