# Supplementary material for: FOXF1 transcription factor promotes lung regeneration after partial pneumonectomy
Source: Sci Rep. 2017 Sep 6;7:10690. doi: 10.1038/s41598-017-11175-3 (PMC5587533; doi:10.1038/s41598-017-11175-3)

FOXF1 transcription factor promotes lung regeneration after partial pneumonectomy.

Craig Bolte[1,2*], Hannah M. Flood[1,2], Xiaomeng Ren[1,2], Artem Barski[3], Jagannathan Sajjeev[3], Tanya V. Kalin[2], Vladimir V. Kalinichenko[1,2*]

[1]Center for Lung Regenerative Medicine, Cincinnati Children's Research Foundation, Cincinnati, Ohio, USA.

[2]Division of Pulmonary Biology, Cincinnati Children's Research Foundation, Cincinnati, Ohio, USA.

[3]Division of Allergy and Immunology, Director of Epigenomics Data Analysis Core, Cincinnati Children's Research Foundation, Cincinnati, Ohio, USA.

***Correspondence** to: Dr. Vladimir V. Kalinichenko (Vladimir.Kalinichenko@cchmc.org) or Dr. Craig Bolte (Craig.Bolte@cchmc.org), Center for Lung Regenerative Medicine, Division of Pulmonary Biology, Cincinnati Children's Hospital Research Foundation, 3333 Burnet Ave., MLC 7009, Cincinnati, OH 45229.

Gene name	Assay number
Ccnd3	Mm01612362_m1
Cd44	Mm01277161_m1
Cdkn1a	Mm04205640_g1
Cdkn2b	Mm00483241_m1
Col1a1	Mm00801666_g1
Col3a1	Mm00802331_m1
Foxf1	Mm00487497_m1
Hdac7	Mm00469527_m1
Mmp14	Mm00485054_m1
Pecam1	Mm01242584_m1
Sox17	Mm00488363_m1
Sftpc	Mm00488144_m1
Spry4	Mm00442345_m1
Timp3	Mm00441826_m1

Supplemental Table 1. List of Taqman probes used in qRT-PCR analysis

Gene	TSS	Binding Sites						
Adamts9	Chr6: 92,901,441	-9727--9869	5308-4898	75129-74923				
Ccnd3	Chr17: 47,505,051	-3284--2886	-1864--1691	-271-599	2956-3150	3373-3538	5781-6659	16799-16942
Cd44	Chr2: 102,901,655	-494--1014	6058-5857	57180-56969	72281-72137			
Cdkn1a	Chr17: 29,093,771	-151-394	1447-1627					
Cdkn2b	Chr4: 89,311,032	897-1073	1866-2032					
Cenpj	Chr14: 56,571,846	-595--452	-288--64					
Flt1	Chr5: 147,725,988	-19162--18975	-11484--10784	18242-18462	23564-23733			
Hdac5	Chr11: 102,230,172	-15282--15651	13879-13534	28512-28307				
Hdac7	Chr15: 97,844,502	-6818--6982	-5580--5833	-5335--5476	3354-3141	13065-12025	23382-22718	30891-30600
		31253-31111						
Itgb3	Chr11: 104,607,999	-18006--17212	3414-4095	6936-7158	9768-10229	14636-15158	18040-18494	32848-33257
		40507-40755						
Itgb4	Chr11: 115,974,725	-9928--9027	-4597--3793	28280-28423				
Kdr	Chr5: 75,978,428	-66836--67420	-9369--9689					
Notch2	Chr3: 98,013,535	-19287--19625	5019-5435	12548-12704	18780-19111	25510-25793	27865-28407	29481-29866
		40235-40732	43281-44062	48295-48669	53592-53764			
Pdgfb	Chr15: 80,014,808	-30792--31301	-9457--9969					
Pecam1	Chr11: 106,715,281	12291-12605	48504-48351	55814-55563				
Ptgs1	Chr2: 36,230,426	-30722--30316	-29650--29233	-16305--16038	3753-4746	16979-17756		
Ptgs2	Chr1: 150,100,124	-2432--2276	-879--584	92-235	5971-6152	7444-7854	7936-8418	
S1pr1	Chr3: 115,715,055	1187-1497						
Spry4	Chr18: 38,601,268	-30692--30112	1553-1860	90810-91267	82852-83415			

Stat1	Chr1: 52,119,437	-6144--6002	-132-151					
Tek	Chr4: 94,739,288	-30059-30202	-15760--15960					
Timp3	Chr10: 86,300,412	-9986--9756	2439-2648	6952-7095	10701-10844	10135-10458	14306-14459	27695-27965
		36701-36987	39295-39438	41933-42092	44983-45256	45876-46135	46954-47097	48314-48500
Tubb4a	Chr17: 57,087,782	-1092--914						

Supplemental Table 2. Genes to which FOXF1 binds as identified by ChIPseq. Sites of FOXF1 binding are listed relative to the transcriptional start site (TSS).

Supplemental Figure 1. FOXF1 present throughout endothelium in regenerating lung.

Immunostaining with FOXF1 antibodies demonstrated that FOXF1 is widely distributed in the alveolar region as well as a subset of endothelium cells in arteries and veins of sham-operated and regenerating lungs. Arrows indicate FOXF1-postive cells. Scale bars are 20 µm. Abbrevations: Br-bronchiole, Ar-artery, Ve- vein.

Supplemental Figure 2. Alveolar size is unaltered during post-PNX regeneration.

(A) Hematoxylin and eosin (H&E) staining of post-PNX lungs from control and *PDGFb-iCre/Foxf1*$^{fl/+}$ mice demonstrate similar morphology. (B) Measurement of air space showed no change in alveolar size following PNX in *PDGFb-iCre/Foxf1*$^{fl/+}$ lungs. (C) Lung tissue resistance was altered by PNX but did not differ between control and *PDGFb-iCre/Foxf1*$^{fl/+}$ mice. (D)Tamoxifen administration did not affect lung regeneration as there was no difference in lung volumes in *Foxf1*$^{fl/+}$ mice with or without tamoxifen or *PDGFb-iCre/Foxf1*$^{fl/+}$ mice in the absence of tamoxifen. (E) qRT-PCR analysis of *Col1a1* and *Col3a1* showed no difference in expression between regenerating control and *PDGFb-iCre/Foxf1*$^{fl/+}$ lungs. (F) Masson's trichrome stain showed no difference in collagen localization between control and *PDGFb-iCre/Foxf1*$^{fl/+}$ mice in regenerating lung. #p<0.05 compared to sham. Scale bars are 20 µm.

Supplemental Figure 3. ChIPseq data shows FOXF1 binding to genes regulating lung regeneration.

Schematic of FOXF1 binding regions in *Adamts9*, *Ccnd3*, *Hdac5*, *Itgb4*, *Ptgs1*, *Ptgs2*, and *Spry4* DNA regulatory regions as determined by ChIPseq. Regions of significant FOXF1 binding to chromatin are show by blue box, transcriptional start sites and direction of translation is shown with a green arrow.

Supplemental Figure 4. Verification of endothelial and epithelial cell populations isolated by FACS.

(A) qRT-PCR analysis of endothelial cells ($CD45^-CD31^+CD326^-$) isolated by FACS. Endothelial cells had high *Pecam-1* expression but did not express surfactant protein C (*Sftpc*). (B) qRT-PCR analysis showed that FACS-sorted epithelial cells ($CD45^-CD31^-CD326^+$) expressed *Sftpc* but not *Pecam-1*.

Supplemental Figure 5. Epithelial cell proliferation in *Foxf1*-deficient mice.

(A) Immunofluorescence experiments were performed using ENDOMUCIN to mark the endothelial network. (B) Mean fluorescent intensity was used to quantify ENDOMUCIN staining. n=4 mice. (C) Co-immunofluorescence experiments using SPC to mark epithelial cells and PCNA to visualize proliferating cells demonstrated epithelial proliferation in control and *PDGFb-iCre/Foxf1*$^{fl/+}$ mice following PNX. (D) No major differences in epithelial proliferation were observed between *PDGFb-iCre/Foxf1*$^{fl/+}$ and control mice during lung regeneration. *p<0.05 compared to controls. Scale bars are 20 µm.

Supplemental Figure 6. *Foxf1*-deficiency does not alter lung apoptosis, low endothelial proliferation in late regeneration.

(A) Immunohistochemistry for Cleaved Caspase 3 showed little apoptosis in control or *PDGFb-iCre/Foxf1*$^{fl/+}$ mice before or after PNX. Scale bars are 20 µm. (B) Histogram and scatter plot showing DNA content as measured using DRAQ5 in FACS-sorted endothelial cells (CD45$^-$CD31$^+$CD326$^-$) from control and *PDGFb-iCre/Foxf1*$^{fl/+}$ lungs 19 days after PNX. (C) Quantification of percentage of endothelial cells in S/G_2/M phases 19 days after PNX in control and *PDGFb-iCre/Foxf1*$^{fl/+}$ lungs. n=3.

Supplemental Figure 7. Mmp14 expression unchanged by *Foxf1*-deficiency.

Western blot analysis showed MMP14 protein levels were not affected by *Foxf1*-deficiency. Cropped gels are presented here with full gel available in Supplemental Figure 9. qRT-PCR analysis showed that *Mmp14* mRNA levels were not altered in *PDGFb-iCre/Foxf1*$^{fl/+}$ lungs compared to control. n=4.

Supplemental Figure 8. Full images for PECAM, CDKN1A, and CDKN2B Western blots.

(A) Full blots for PECAM-1 and corresponding β-ACTIN along with digital image of blot showing ladder. PECAM-1 Western blot using Abcam 28364 antibody has been previously published [1]. Western blot using Santa Cruz β-ACTIN antibody SC-1615 has also been previously published[2]. (B) Full blots for CDKN1A (P21^{Cip1}), CDKN2B (P15^{INK4b}), and corresponding β-ACTIN. Membrane was cut prior to staining so ladder is not shown. CDKN1A Western blot using Cell Signaling Technology antibody 2947 has been previously published[3]. CDKN2B Western blot using Aviva Systems Biology antibody AVARP03047 has been previously published[4].

Supplemental Figure 9. Full images for Western blots and zymography.

(A) Full zymography gel used for analysis of MMP14 activity. (B) Full blots for MMP14 and corresponding β-ACTIN. MMP14 Western blot using Abcam 51074 antibody has been previously published[5].

Supplemental References

1 Corallo, C. *et al.* Bosentan and macitentan prevent the endothelial-to-mesenchymal transition (EndoMT) in systemic sclerosis: in vitro study. *Arthritis Res Ther* **18**, 228, doi:10.1186/s13075-016-1122-y (2016).

2 Sato, M. *et al.* LW6, a hypoxia-inducible factor 1 inhibitor, selectively induces apoptosis in hypoxic cells through depolarization of mitochondria in A549 human lung cancer cells. *Mol Med Rep* **12**, 3462-3468, doi:10.3892/mmr.2015.3862 (2015).

3 Fiorentino, F. P. *et al.* Growth suppression by MYC inhibition in small cell lung cancer cells with TP53 and RB1 inactivation. *Oncotarget* **7**, 31014-31028, doi:10.18632/oncotarget.8826 (2016).

4 Scott, S. A. *et al.* Methylation status of cyclin-dependent kinase inhibitor genes within the transforming growth factor beta pathway in human T-cell lymphoblastic lymphoma/leukemia. *Leuk Res* **28**, 1293-1301, doi:10.1016/j.leukres.2004.03.019 (2004).

5 Ding, B. S., Gomi, K., Rafii, S., Crystal, R. G. & Walters, M. S. Endothelial MMP14 is required for endothelial-dependent growth support of human airway basal cells. *J Cell Sci* **128**, 2983-2988, doi:10.1242/jcs.168179 (2015).

Bolte Supplemental Figure 1

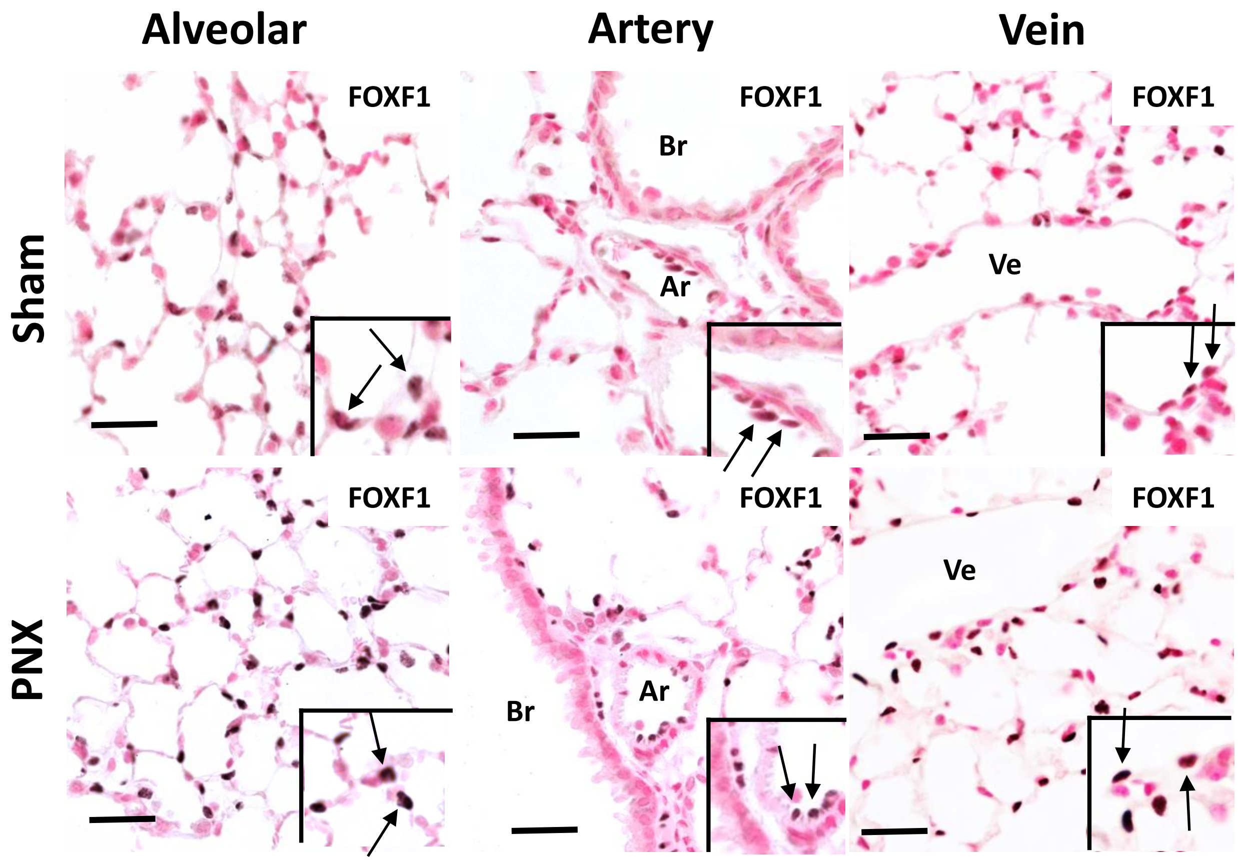

Bolte Supplemental Figure 2

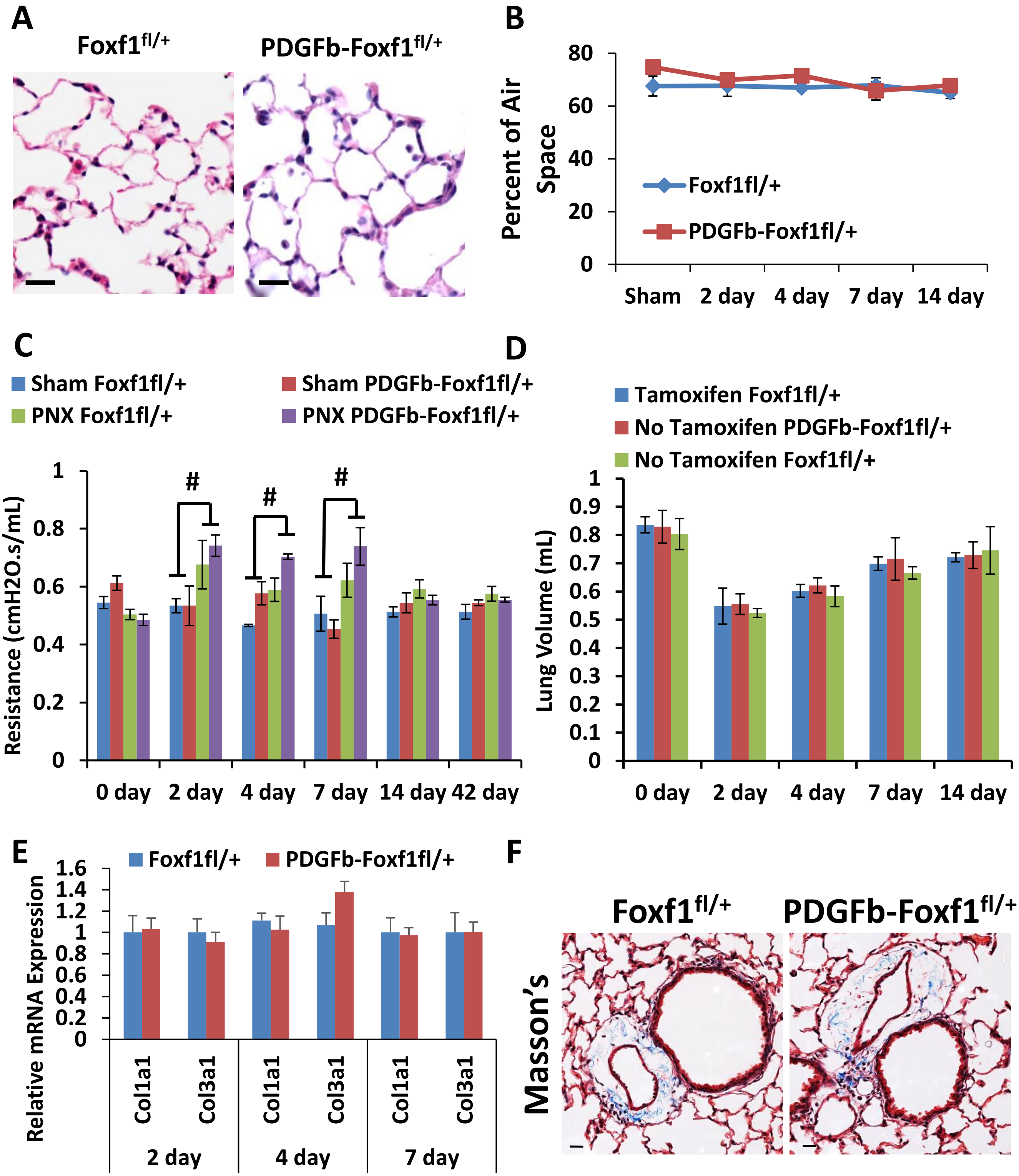

Bolte Supplemental Figure 3

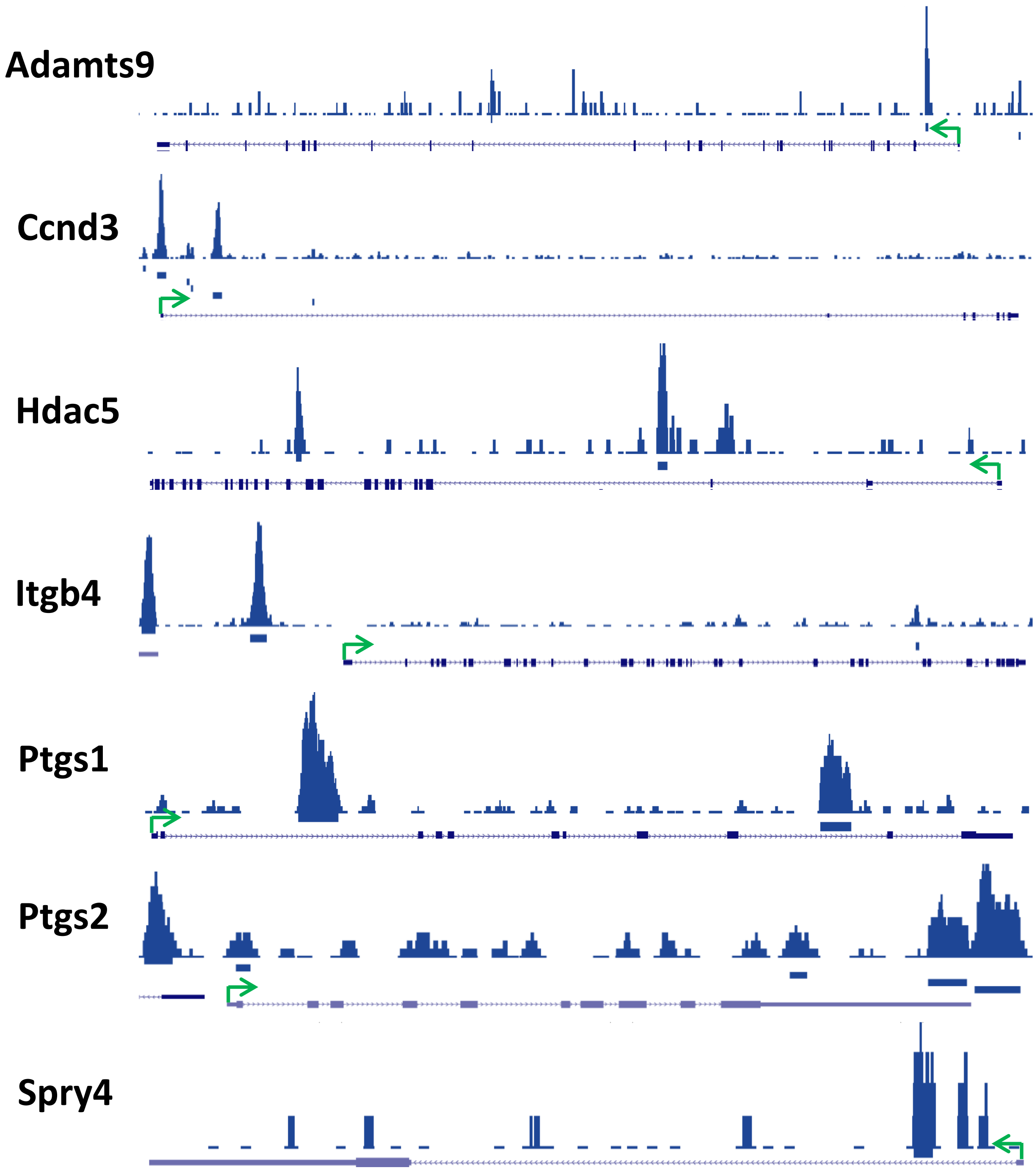

Bolte Supplemental Figure 4

A

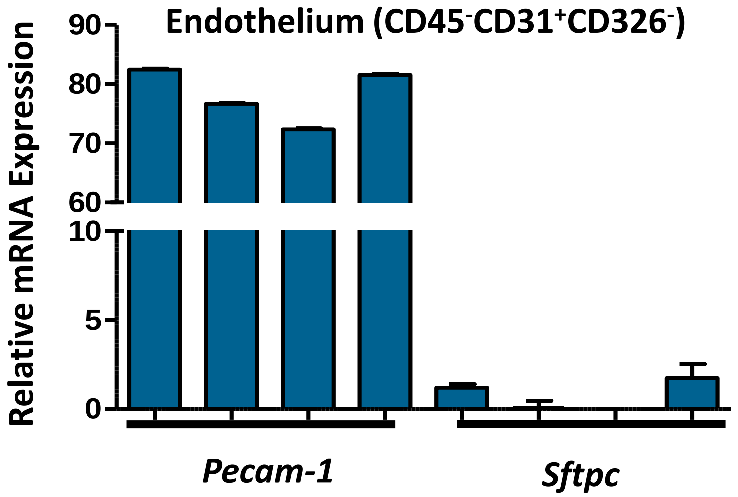

B

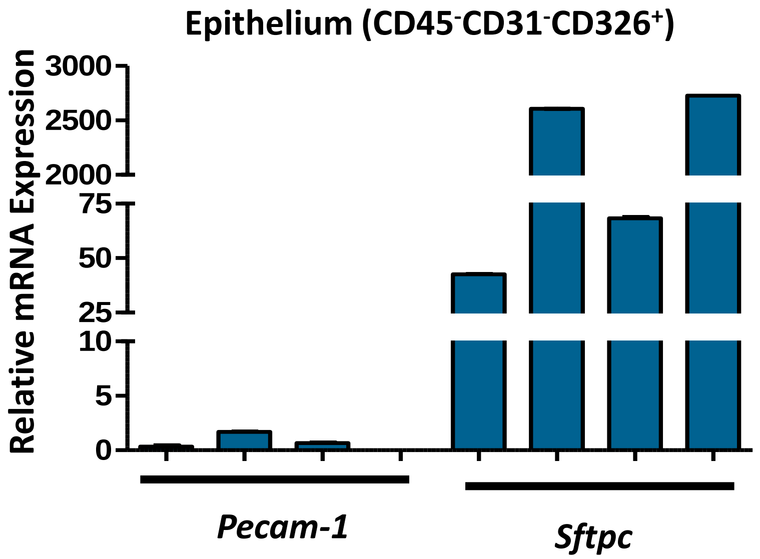

Bolte Supplemental Figure 5

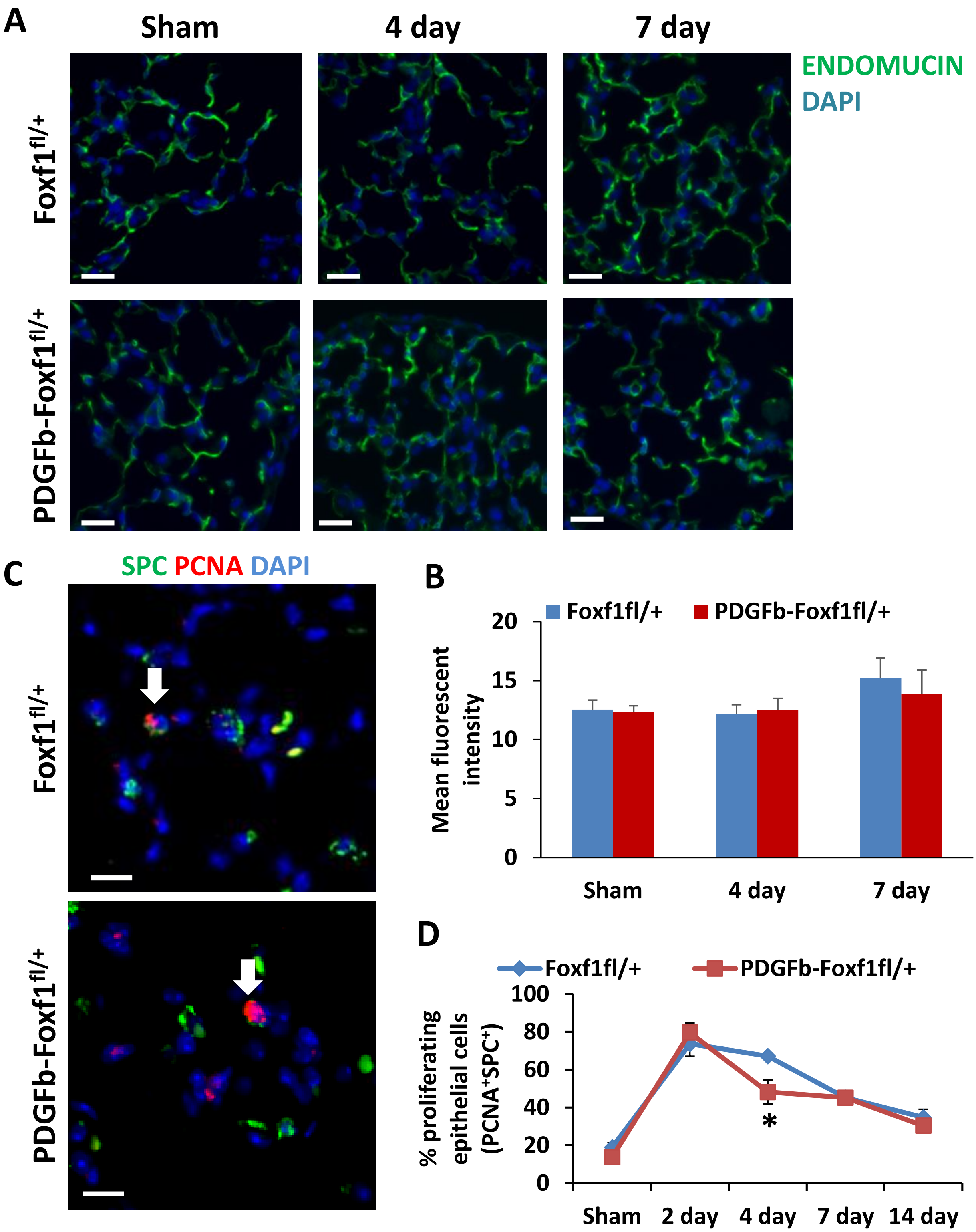

Bolte Supplemental Figure 6

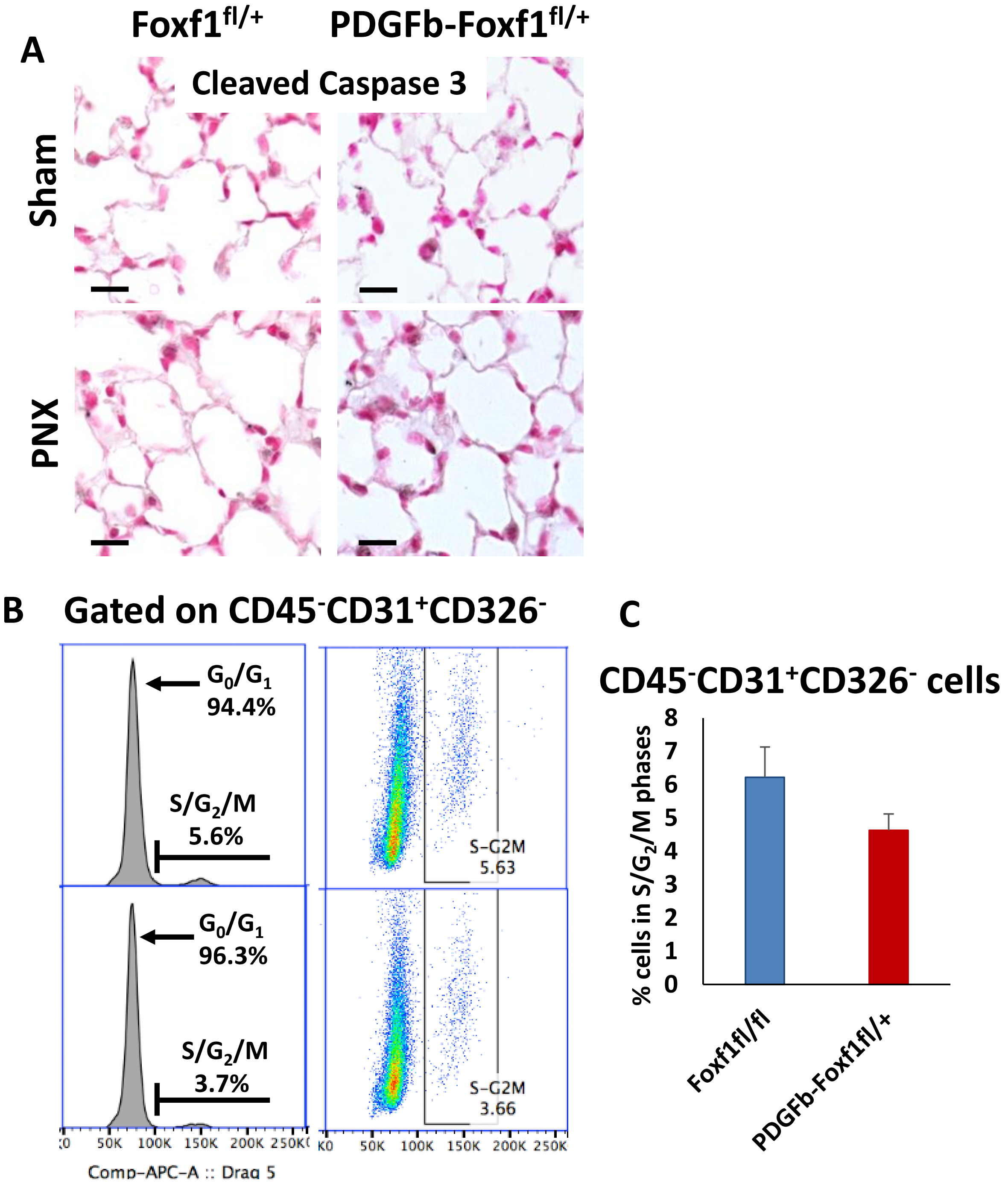

Bolte Supplemental Figure 7

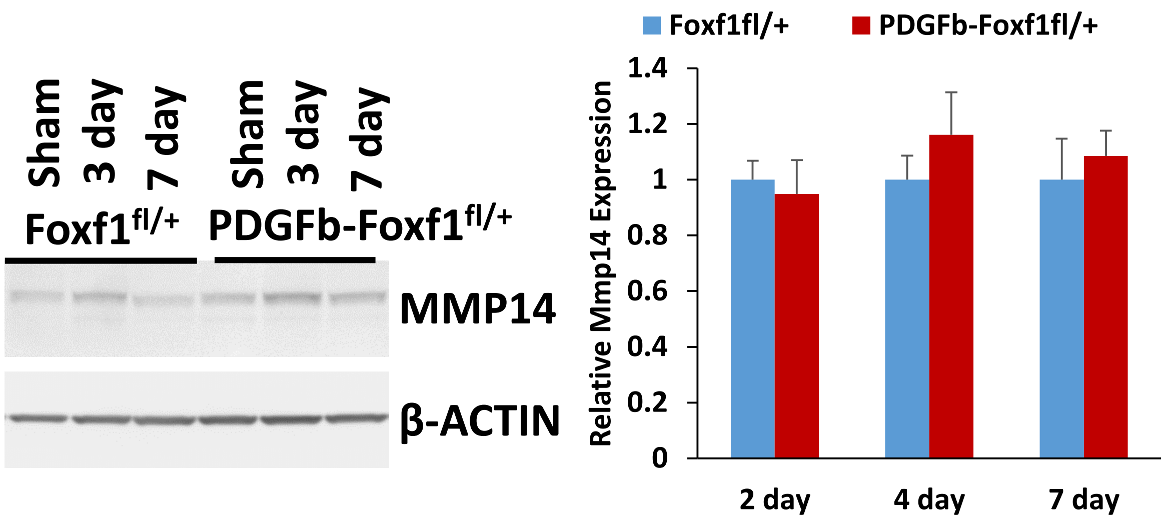

Bolte Supplemental Figure 8

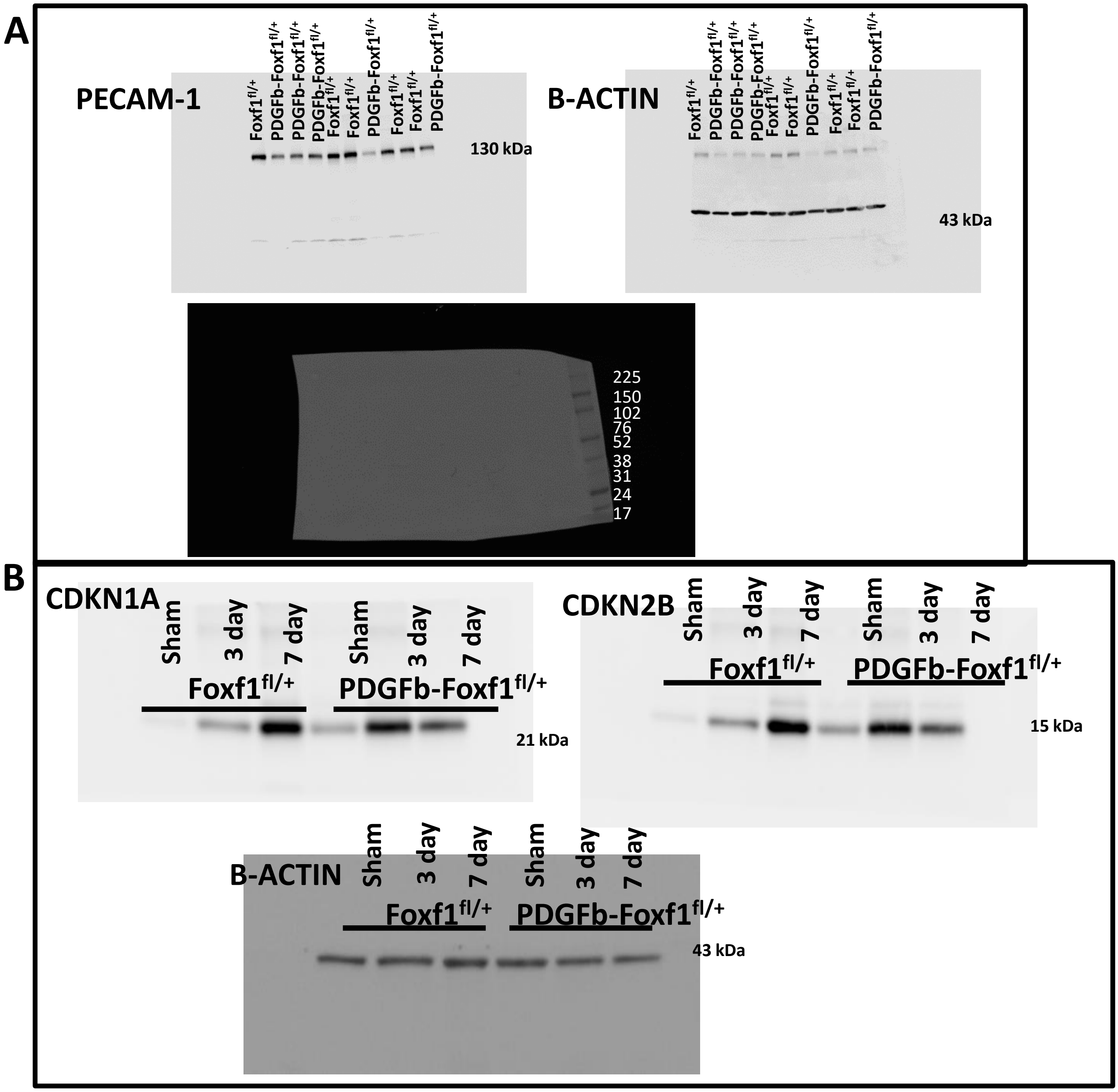

Bolte Supplemental Figure 9

A

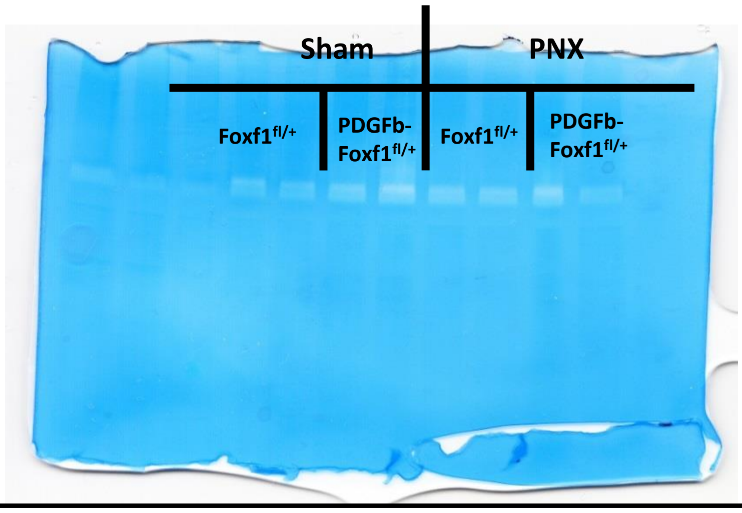

B

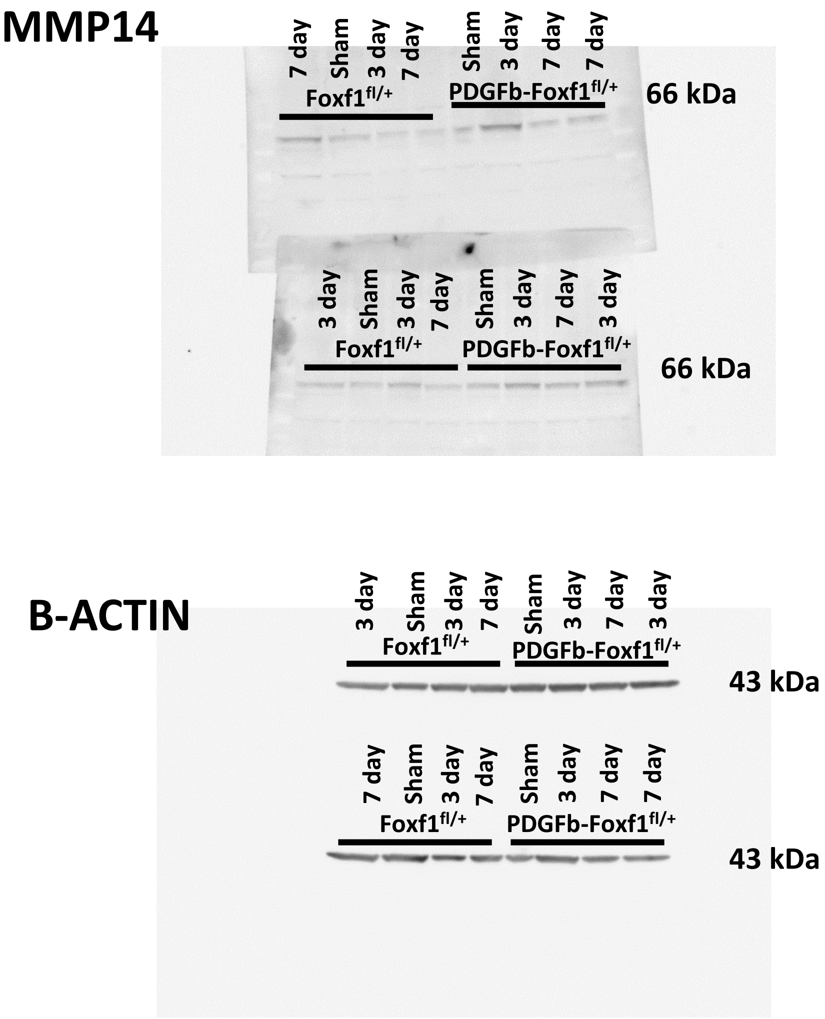

Supplement: Supplementary file 1 — All supplemental files [file 41598_2017_11175_MOESM1_ESM.pdf]
